# Adsorption of extracellular vesicles onto the tube walls during storage in solution

**DOI:** 10.1371/journal.pone.0243738

**Published:** 2020-12-28

**Authors:** Evgeniy G. Evtushenko, Dmitry V. Bagrov, Vassili N. Lazarev, Mikhail A. Livshits, Elena Khomyakova

**Affiliations:** 1 Department of Chemical Enzymology, Faculty of Chemistry, Lomonosov Moscow State University, Moscow, Russian Federation; 2 Department of Bioengineering, Faculty of Biology, Lomonosov Moscow State University, Moscow, Russian Federation; 3 Federal Research and Clinical Center of Physical-Chemical Medicine of Federal Medical Biological Agency, Moscow, Russian Federation; 4 Moscow Institute of Physics and Technology (State University), Dolgoprudny, Russian Federation; 5 Engelhardt Institute of Molecular Biology, Russian Academy of Sciences, Moscow, Russian Federation; The Ohio State University, UNITED STATES

## Abstract

Short term storage of extracellular vesicle (EV) solutions at +4°C is a common practice, but the stability of EVs during this procedure has not been fully understood yet. Using nanoparticle tracking analysis, we have shown that EVs isolated from the conditioned medium of HT-29 cells exhibit a pronounced concentration decrease when stored in PBS in ordinary polypropylene tubes within the range of (0.5–2.1) × 10^10^ particles/ml. EV losses reach 51±3% for 0.5 ml of EVs in Eppendorf 2 ml tube at 48 hours of storage at +4°C. Around 2/3 of the observed losses have been attributed to the adsorption of vesicles onto tube walls. This result shows that the lower part (up to at least 2 × 10^10^ particles/ml) of the practically relevant concentration range for purified EVs is prone to adsorption losses at +4°C. Total particle losses could be reduced to 18–21% at 48 hours by using either Eppendorf Protein LoBind tubes or ordinary tubes with the surface blocked with bovine serum albumin or EVs. Reduction of losses to 15% has been shown for isolated EVs dissolved in the supernatant after 100 000 g centrifugation as a model of conditioned medium. Also, a previously unknown feature of diffusion-controlled adsorption was revealed for EVs. In addition to the decrease in particle count, this process causes the predominant losses of smaller particles.

## Introduction

It has been well established that mammalian cells release various types of lipid bilayer surrounded particles generally named extracellular vesicles (EVs) [1,2]. Being involved in a wide range of physiological and pathological processes, EVs were extensively studied over the past few decades [3–5]. Since the beginning of the rapid expansion of EVs related research in the late 1990s and early 2000s, many efforts were devoted to the development of proper and standardised protocols for EVs isolation, characterisation, handling, etc. [6–9]. Despite the significant progress in this field, some important aspects of EVs behaviour are still not well understood. One such case is the storage or handling of EVs solutions at temperatures above 0°C, i.e., without freezing.

The consensus about the proper conditions for long term storage of EVs from days to months is freezing at -80°C or below [6,8]. Short term storage from few hours to few days is often performed at +4°C, and some papers report doing so [10–12]. These storage conditions are common in laboratory practice, most likely, due to a general viewpoint of extreme stability of EVs as well as reports of EV degradation during repeated freeze/thaw cycles (see review [13] for detailed discussion). Besides storage, many experimental procedures require incubations of EV containing solutions at +4°C for several hours, for instance, specific binding of EVs to beads, uptake of EVs by cells, etc.

Several studies were dedicated specifically to EVs stability at +4°C or room temperature or contain large sections devoted to this topic [14–28]. Additional pieces of information could be found in research papers, where the EVs stability has been checked [29–37] in line with the general topic. All these data were summarised in S1 and S2 Tables. This dataset is highly heterogeneous as it contains both quantitative and semi-quantitative results, as well as qualitative observations with different parameters of EVs tested as a measure of stability. Also, these data might be biased because the results obtained with unstable EVs are less likely to be published. Nevertheless, the following pattern might be proposed based on these data. (1) EVs tend to be stable (10 out of 13 studies) being stored in complex media (blood plasma or serum, urine, saliva, EVs isolated with ExoQuick™ or similar kits and containing large amounts of polymer and some entrapped protein) at +4°C and in some cases even at +25°C. (2) Purified EVs isolated by ultracentrifugation, or gradient centrifugation, or chromatography and resuspended in phosphate-buffered saline (PBS) are likely to be unstable when stored at +4°C (7 out of 8 studies), exhibiting either particle concentration decrease, or lowering of specific marker levels, or shift in particle size distribution (PSD).

Practical recommendations from protocols also vary. Precleared ovarian follicular fluids might be kept at +4°C for up to a week prior to EVs isolation [38]. The storage at +4°C of purified EVs for up to 48 hours is allowed by protocol [39] (isolation by size-exclusion chromatography) and for 72 hours by protocol [40] (isolation by ultracentrifugation), while [41] recommend keeping isolated EVs on ice and process them as soon as possible. This ambiguity indicates the necessity of careful examination of the EVs instability phenomenon at +4°C.

**Fig 1:**
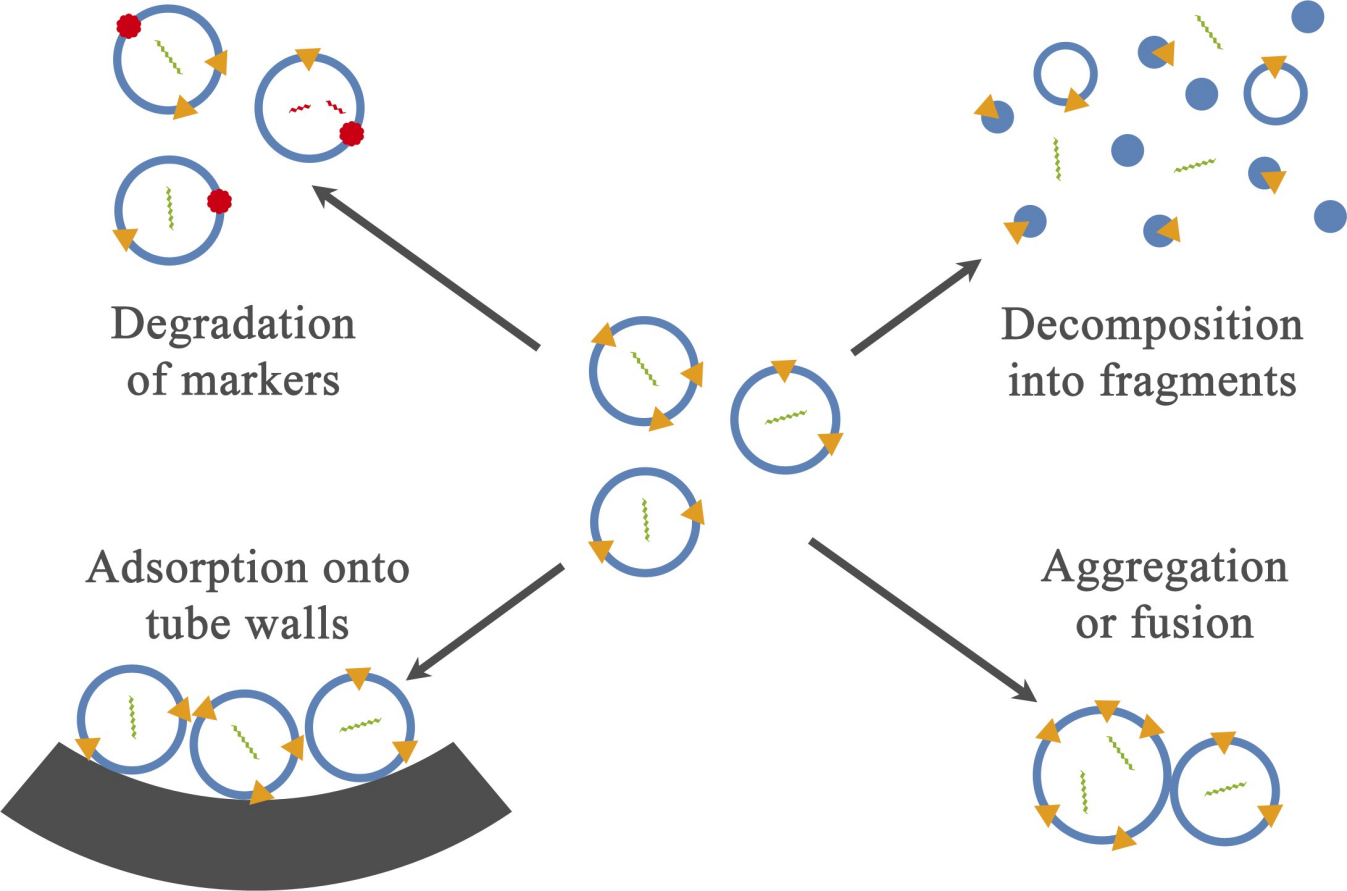
Hypothetical routes of EVs losses/degradation during storage at +4°C proposed in the literature.

Several distinct processes hypothetically might be responsible for the degradation of purified EVs with time at +4°C (Fig 1):

- Degradation of EV’s proteins [16,17] or other marker molecules occurred without changes in vesicles’ integrity or count. This hypothesis contradicts reports claimed particle concentration decrease [18,25] and changes in PSD [14,34]. On the other hand, this process may accompany other degradation routes [25].
- Decomposition of vesicles into smaller fragments [34] accompanied by leakage of their content into the surrounding solution. This route is supported by a decrease in mean particle size during long term storage of EVs at +4°C [14].
- Aggregation [12,13] or fusion [17,25] of vesicles. It is hard to distinguish between these two processes, but both of them reduce the particle count and increase the mean size.
- Adsorption of vesicles onto vessel walls. No direct experimental evidence for this route was reported for EVs in peer-reviewed papers, but multiple researchers warned that this process might occur during storage [6,20,33,42]. Some studies and protocols reported the usage of low protein binding tubes for EVs storage or handling [32,41,43–45] with no experimental basis for this choice. Recently a patent for hydrophilic polymeric coating/additive to prevent EV adsorption was published [46] together with commercial EV-Save™ blocking reagent (058–09261, FUJIFILM Wako Pure Chemical Corporation). Both the patent and the reagent technical information report substantial losses of EV during isolation and handling due to adsorption. At the same time, this reagent has not been used in any published papers so far.

Proposed mechanisms should exhibit different behaviours if initial EVs concentration is varied. A process of marker molecule degradation without changes in particle count is independent of particle concentration. Three remaining mechanisms cause a decrease in concentration with time. Relative losses of vesicles in a case of their decomposition into smaller fragments should be independent of initial concentration as every vesicle decomposes on its own. The early stage of aggregation/fusion, on the other hand, is a process between two particles, so the relative losses for any given time should rise with the increase in initial concentration. Adsorption of EVs onto the surface of the tube behaves differently. If the concentration is high and the total amount of substance is much larger than surface binding capacity, almost no relative losses occur. If the concentration is low enough, so the total amount of substance is low compared to surface binding capacity, everything will eventually be lost from the solution.

Thus, the present study aims to investigate processes occurring during EV solutions short term storage at +4°C to provide further insight into underlying mechanisms of the EV losses.

## Materials and methods

### Cells

The HT-29 human colorectal adenocarcinoma cell line (ATCC HTB-38) was grown in culture flasks to 80–90% confluence in Dulbecco′s Modified Eagle′s Medium (DMEM, Sigma, USA) supplemented with 10% fetal bovine serum (FBS, Gibco, USA), 2 mM glutamine, 5 μg/ml gentamicin at 37°C and 5% CO_2_. Cells were washed three times with phosphate-buffered saline (PBS, Gibco, USA) and conditioned in serum-free DMEM for 24 hours.

### Isolation of EVs from the conditioned medium by differential centrifugation

Isolation of EVs was performed according to the previously described procedure [47]. Briefly, the cell culture medium was successively centrifuged at +4°C for 10 min at 300 g, 10 min at 2 000 g (A-4-44 swing-bucket rotor, Eppendorf AG, Germany) and 30 min at 10 000 g (Type 60 Ti fixed angle rotor, Beckman Coulter, USA) to pellet cells, debris, and large vesicles correspondingly. The supernatant was centrifuged for 70 min at 100 000 g at +4°C in Type 60 Ti rotor. The pellet was resuspended in PBS. The supernatant (further referred as ‘supernatant after 100 000 g’) was collected for storage experiments.

### Particle size distribution and concentration measurements

Particle size distributions and particle concentrations were measured with Nanoparticle Tracking Analysis (NTA) using Nanosight LM10 HSBF instrument (Nanosight Ltd, UK). The configuration contained 405 nm, 65 mW laser unit with passive temperature readout and high sensitivity camera Andor Luca of EMCCD type. NTA 2.3 build 33 software (Nanosight, UK) was used. All measurements were performed according to ASTM E2834 - 12(2018) recommendations [48]. Samples were diluted 30–3000 times by particle-free PBS to reach the concentration (0.8–1.3)×10^8^ particles/ml. Eighteen (in rare cases, 12 or 15) videos, 60 s each, were recorded using following camera settings in advanced mode: Shutter = 850, Gain = 450, Lower threshold = 910, Higher threshold = 11180. Processing was performed in basic mode using the following setups: Detection threshold = 9 Multi, Min expected size = 30 nm. These settings were previously optimised for EVs measurements using the particular instrument configuration [47,49]. Particles from all recorded videos were collected in a single table (3700–8100 total tracks) followed by calculation of joined PSD histogram, mean size, and total particle concentration, corrected for dilution factor.

### Transmission Electron Microscopy (TEM)

The carbon-coated TEM grids (Ted Pella, USA) were treated using a glow discharge device Emitech K100X (Quorum Technologies, UK) to hydrophilise the carbon surface and increase the adsorption. The exosomes were deposited onto the grids for 3 min, contrasted with 1% uranyl acetate, and dried. Imaging was carried out using a JEM-1011 (Jeol, Japan) transmission electron microscope at 80 kV.

### EVs storage protocol

Freshly isolated EVs from a single batch were measured by NTA, aliquoted, frozen at -80°C and stored no longer than 8 weeks. Several aliquots were thawed, mixed together, diluted to desired initial concentration by the storage medium (PBS, or serum-free, particle-free DMEM, or supernatant after 100 000 g centrifugation), divided into 5 portions 0.5 ml each, and placed into 5 identical tubes. Following types of tubes were used: ordinary Eppendorf Safe-Lock 2 ml (0030 120.094, Eppendorf AG, Germany), or Eppendorf Safe-Lock 2 ml treated with bovine serum albumin (BSA) or EVs, or Eppendorf Safe-Lock Protein LoBind 2 ml (0030 108.132), or Axygen 2 ml tubes (MCT-200-C, Corning, USA), or Axygen Maxymum Recovery (MCT-200-L-C) 2 ml tubes. All five portions of EVs in identical tubes were immediately placed to +4°C and stored for 0.5, 6, 12, 24, and 48 hours correspondingly. In order to avoid the influence of mixing during aliquots withdrawal for NTA measurements, any single portion was used for one particular time (e.g., tube #1 –for 0.5 h measurements) and then discarded. As long as full NTA measurement takes around 1 h, reported mean size and total concentration are the average values between -0.5 h and +0.5 h relative to indicated storage duration (e.g. ‘concentration at 0.5 h’ means the average concentration between 0 and 1 hour of storage; ‘mean size at 12 h’ means the average mean size between 11.5 and 12.5 h of storage). Exact initial concentrations for ‘×1’ samples was measured for freshly thawed sample at zero storage time in ordinary Eppendorf tubes as (1.03±0.07)×10^10^ (N = 36), (1.02±0.09)×10^10^ (N = 18), and (1.09±0.08)×10^10^ (N = 24) particles/ml for EV batches 1, 2, and 3 correspondingly. Initial concentrations for ‘×0.5’, ‘×0.75’, and ‘×2’ samples were calculated using dilution factor ratios. Normalised values and corresponding losses were calculated as a ratio between measured concentrations and the initial ones.

BSA-blocked Eppendorf Safe-Lock 2 ml tubes were prepared by incubation of 10 mg/ml of bovine serum albumin (Sigma, min 98%) in PBS for 1 h at room temperature, followed by 5× wash by particle-free PBS. EV-blocked Eppendorf Safe-Lock 2 ml tubes were prepared by incubation of 2×10^10^ particles/ml of EVs in PBS for 72 hours at +4°C followed by 5× wash by particle-free PBS. Both BSA- and EV-blocked tubes were checked by NTA for the presence of adventitious nanoparticles (protein aggregates of EVs desorbed from the surface) and used immediately after the preparation.

### Numerical simulation of the diffusion-adsorption process

The diffusion-adsorption problem for experimental geometry (0.5 ml of solution in 2 ml Eppendorf tube with an inner diameter of 0.88 cm) was numerically simulated using the Fick’s second law of diffusion by finite elements method in Mathematica 10.2 package (Wolfram Research, USA). The case of unlimited and instantaneous adsorption was used with C_wall_ = 0 at any time. In order to evaluate the applicability of the model to adsorption with monolayer saturation, the total flux over the solution-wall boundary was calculated at 48 h and checked to be below the maximum capacity of the tube wall. Further details of the simulation are described in the S1 Appendix.

### Statistical analysis

All calculations of statistical parameters were performed using the built-in functions of Mathematica 10.2 package. Confidence intervals (CI) for mean values in replicate NTA measurements were estimated using Student t-distribution with a 95% confidence level (MeanCI function). A two-sample t-test was used for sample comparison. Usage of paired (PairedTTest function) or unpaired (TTest function) test is explicitly indicated for each case. One-way ANOVA was used to compare more than two groups (ANOVA function). Differences were considered significant at *p* ≤ 0.05.

## Results and discussion

### Characterisation of isolated EVs

**Fig 2:**
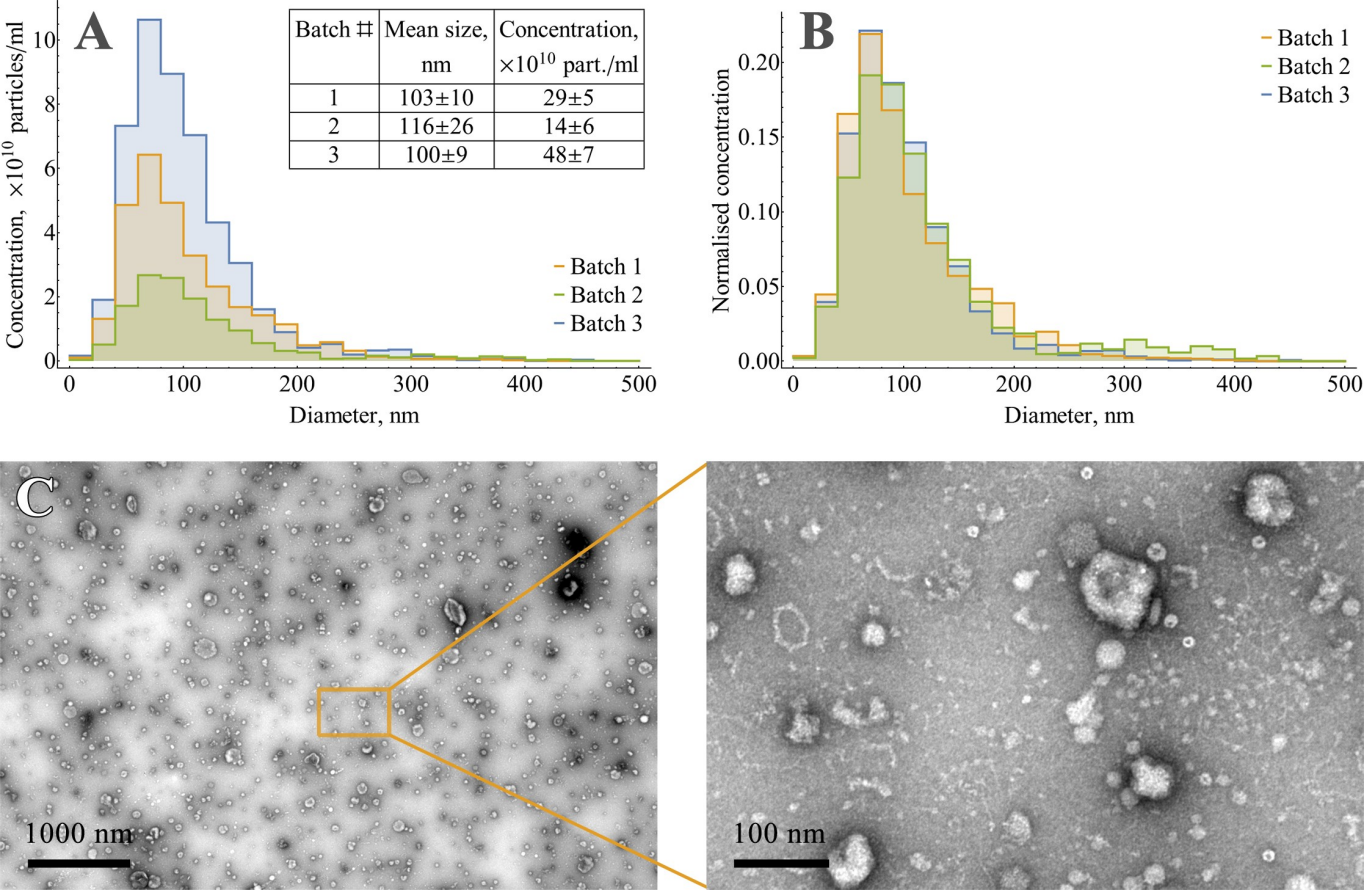
Characterisation of isolated vesicles from HT-29 conditioned medium. (A) NTA mean sizes and concentrations for three independent EV batches. Errors represent 95% CI of the mean (N = 12). (B) Normalised particle size distributions for three independent batches of EVs. (C) TEM of isolated vesicles.

Analysis of PSD, mean size, and total particle concentration for 3 batches of EVs (independent cell cultivations, cell culture medium harvesting, and EV isolation) have shown that particles slightly varied in mean size and might differ up to 3.5 times in concentration (Fig 2A). At the same time, normalisation by concentration confirmed consistency in PSD between batches (Fig 2B). As long as +4°C stability measurements were made with EVs, previously stored at -80°C, changes of mean size and PSD after freeze/thaw procedure were also evaluated (S1 Fig) and shown to be minor.

TEM images of isolated EVs indicated the typical for extracellular vesicle morphology and size range (Fig 2C).

### Purified EVs stored in PBS at +4°C exhibit pronounced particle losses at concentrations up to 2×10^10^ particles/ml

**Fig 3:**
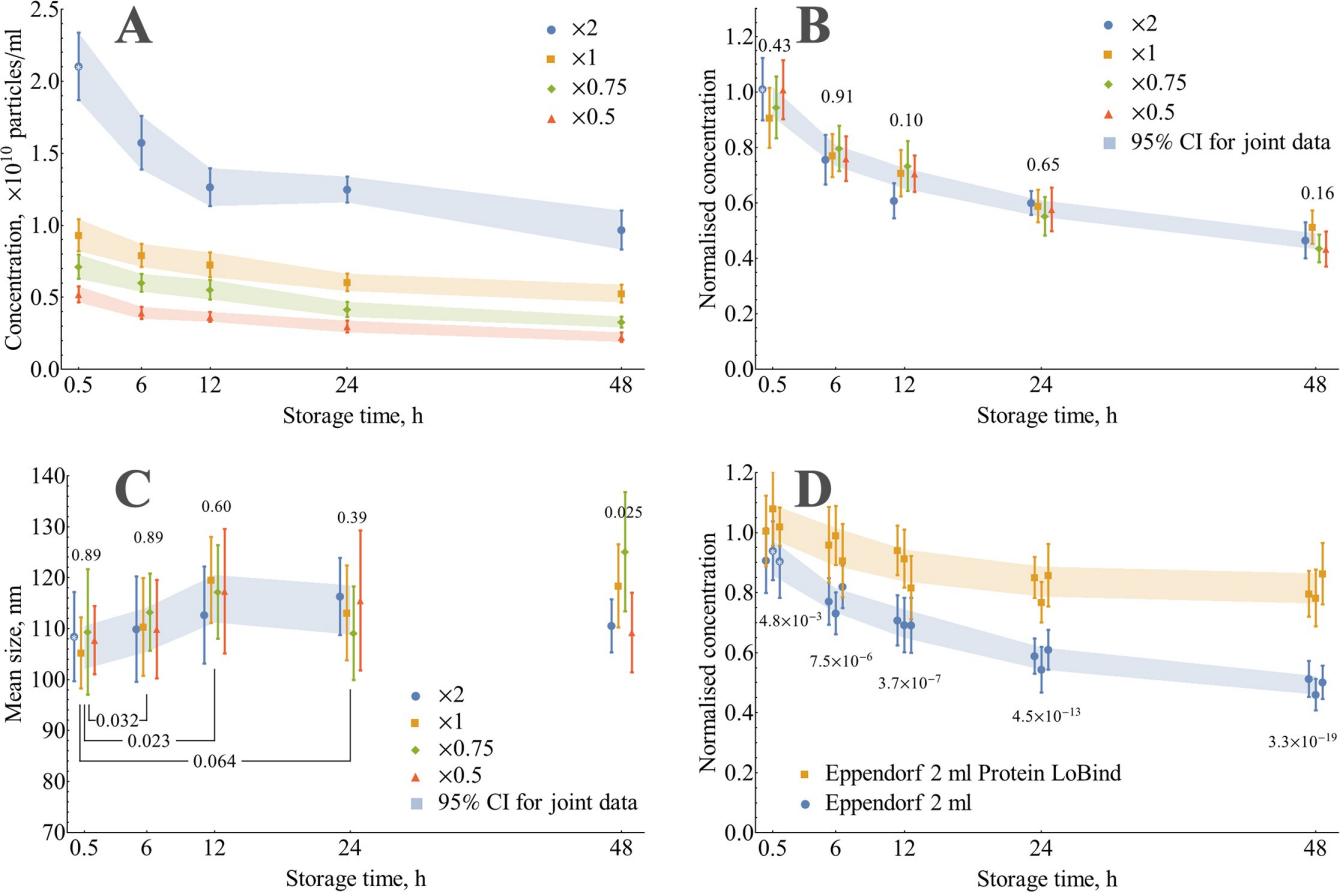
Evolution of particle concentration and mean size during the storage of isolated EVs in PBS (0.5 ml) at +4°C. (A) Concentration changes for the same batch of EVs at different initial concentrations in ordinary Eppendorf 2 ml tubes. (B) Data of panel A normalised by initial vesicle concentration. The numbers above groups indicate ANOVA *p*-values for each storage time. (C) Mean size changes for the same samples. The numbers above groups indicate ANOVA *p*-values for each storage time. *P*-values for differences with 0.5 h were calculated using a two-tailed paired t-test. (D) Evolution of normalised particle concentration for three independent EV batches during storage in PBS (initial concentration 1×10^10^ particles/ml) in ordinary Eppendorf 2 ml tubes and Eppendorf Protein LoBind 2 ml tubes. Numbers below groups indicate *p*-values of a two-tailed unpaired t-test between the common tube and LoBind one. Error bars for individual data points on each panel represent 95% CI of the mean (N = 12 for points marked with an asterisk and N = 18 for the rest).

It has been proposed earlier from the literature review that the EVs instability phenomenon was most likely to occur for purified vesicles. In order to check the stability for purified EVs from HT-29 cells, a 48 h storage at +4°C in PBS with measurements of both size and concentration evolution was performed. Samples from the single EV batch diluted to different initial concentrations were tested: 0.52, 0.76, 1.0, and 2.1×10^10^ particles/ml. A pronounced decrease in concentration was observed for all tested samples (Fig 3A). Normalisation by initial concentration showed that relative losses for any measured storage time were similar despite the 4-fold span in initial concentrations (Fig 3B and S2 Fig). Size increase has been detected for the first 12 h; however, the samples diverged in size during further storage (Fig 3C).

A preliminary ranking of the likelihood of proposed losses mechanisms might be performed based on these data. The observed particle concentration decrease contradicts marker degradation as the standalone phenomenon, because it implies no changes in concentration; however, it might accompany other routes. Size increase during the first 12 h of storage does not support the hypothesis of vesicles decomposition into smaller fragments as the main route. Independence of relative losses on initial concentration makes aggregation/fusion a less likely cause despite the increase in size. Adsorption onto the tube walls might explain concentration decrease, but size evolution interpretation is not straightforward based on this data.

### Storage of purified EVs in Eppendorf Protein LoBind tubes reveals the significant role of adsorption during 2 days of storage at +4°C

In order to directly evaluate the impact of adsorption on the kinetics of concentration losses, it was compared by NTA for two types of tubes of identical geometry: ordinary Eppendorf 2 ml and Eppendorf Protein LoBind tubes 2 ml (Fig 3D and S3 Fig). Concentration drop of 51±3% at 48 h storage time in ordinary tubes was far larger than the 19±5% losses in Protein LoBind tubes. It indicates the massive impact of adsorption on concentration decrease for studied concentration range: at least 32% loses out of total 51% (approximately 2/3) should be attributed to adsorption of vesicles onto polypropylene tube walls.

Protein LoBind tubes were designed to prevent protein adsorption, but EVs also have lipid bilayer areas on their surface. Thus, some fraction of losses in these tubes might also be caused by adsorption, and the 32% difference between ordinary tubes and LoBind ones should be considered as a lower bound for adsorption losses. Hereinafter we shall refer to this difference as ‘proved adsorption losses.’

### Observed magnitude and kinetics of vesicle losses in ordinary tubes do not contradict theoretical considerations

Although we have experimentally shown the dominance of adsorption on tube walls over other routes of EV losses for tested concentration range, it might be useful to verify our findings. It has been done both in terms of (a) magnitude (i.e., maximum observed relative losses) by theoretical considerations of wall binding capacity and (b) kinetics (relative losses for every measured time) by numerical simulation of the diffusion-adsorption process.

The theoretical magnitude of adsorption-driven losses has been estimated by the calculation of the maximum binding capacity of the ordinary tube wall being in contact with the solution. The total geometric area of the wall-solution boundary for 2 ml Eppendorf tube and 0.5 ml of the solution might be estimated as 2.7 cm^2^ using tube dimensions provided by the manufacturer (S1 Appendix). Using atomic force microscopy measurements (S2 Appendix), we have shown that the difference between the real surface area and the geometric one for ordinary Eppendorf tubes does not exceed 1.7%. The tightest way to fit a maximum number of vesicles on a given area is a hexagonal packing arrangement, where every vesicle of diameter *d* occupies the surface area of 32×d2. The largest area occupied by a single vesicle might be estimated as *π*×*d*^2^ for the hypothetical process of supported lipid bilayer formation, which is well known for liposomes [50] and also has been shown to occur for EVs on mica modified by 3-aminopropyltriethoxysilane [51]. Using these bounds, the estimated binding capacity of the tube wall for 0.5 ml of the solution containing 100 nm vesicles ranges from 0.9 to 3.1 × 10^10^ particles. These values correspond to a maximum possible concentration decrease for 0.5 ml of solution ranging from 1.7 to 6.2 × 10^10^ particles/ml. The highest observed concentration drop of 1.1 × 10^10^ vesicles/ml (sample ‘×2’ in Fig 3A) is lower than this estimate. Thus, the magnitude of the observed concentration losses for ordinary tubes does not contradict the theoretical considerations.

According to storage protocol, the samples were stored at +4°C undisturbed, i.e., there was no mixing starting from tube filing up to NTA measurement of the sample at desired storage time. In these conditions, diffusion might be considered as the primary mechanism of particles’ transfer from inner parts of the solution to tube walls. This problem of diffusion-controlled adsorption has been numerically solved for exact experimental storage geometry (0.5 ml of solution in 2 ml Eppendorf tube). The model of unlimited adsorption was used as we have previously shown that maximum binding capacity has not been reached in any of our experiments with ordinary tubes.

**Fig 4:**
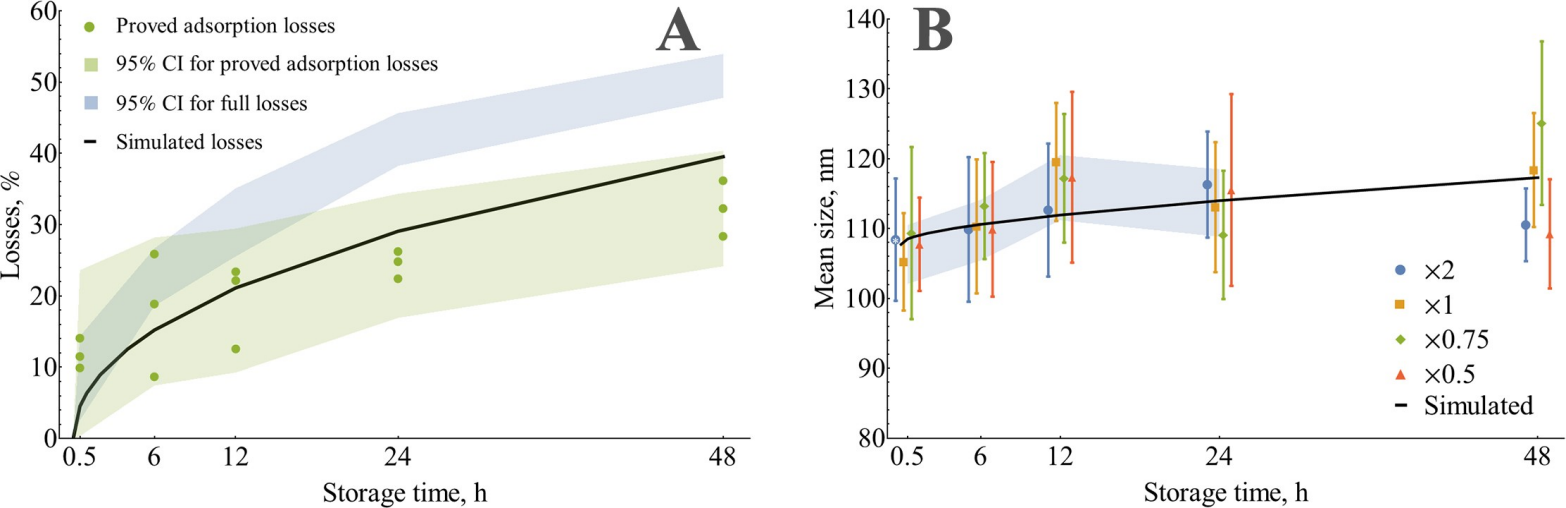
Comparison of measured adsorption kinetics and numerical simulation. (A) Evolution of simulated adsorption losses compared to full losses (PBS, ordinary Eppendorf tube) and proved adsorption losses (PBS, the difference between ordinary Eppendorf tube and Protein LoBind one). (B) Measured size evolution for different initial concentrations compared to simulated size changes. Error bars for individual data points represent 95% CI of the mean (N = 12 for points marked with an asterisk and N = 18 for the rest).

The diffusion equation, together with initial and boundary conditions, is linear in concentration (S1 Appendix). It means that for unlimited adsorption, i.e., when the initial concentration is far below the maximum concentration drop, relative losses at any given time do not depend on initial concentration in an agreement with kinetics, measured by NTA. Simulated relative losses (Fig 4A) reach 40% for a storage time of 48 h. This value exceeds the proved adsorption losses (32%) but smaller than the total losses (51%), thus does not contradict experimental data.

The diffusion-controlled adsorption also explains the effect of mean size increase. Smaller particles with higher diffusion coefficients are transferred to the wall faster than larger ones. Thus, the mean size of the remaining particles increases. Using the same numerical model, we calculated the evolution of mean size during storage, assuming that the initial PSD is known from NTA measurements (Fig 2B). The result of this simulation (Fig 4B) shows a 9.6 nm increase in size at 48 h in good agreement with the measured size evolution.

Thus, theoretical considerations and numerical simulation confirmed that observed changes are fully consistent with the adsorption mechanism of losses. It should also be noted that the simulation revealed the important features of the adsorption process occurred under diffusion control (i.e., without mixing). Adsorption-driven changes in size and concentration are very similar to aggregation/fusion; both processes cause a decrease in particle count and increase in size. Also as long as adsorption losses under diffusion control are size-specific and smaller vesicles show greater losses, this process enriches the sample with larger particles represented by microvesicles fraction. This effect might potentially alter the results of any study of vesicle cargo: miRNA, proteomic, etc.

### Comparison of observed effects to published data

Direct comparison of our findings to published data is not possible since the role of adsorption during the storage of EVs has not been studied previously. Nevertheless, we could compare our experimental data on the size and concentration trends to published studies [14,17,18,25,34]. It is not straightforward, because only one study [25] contains a full description of storage protocol (volume of solution and concentration of vesicles, test tube used for storage, whether aliquots for each storage duration were taken from the same tube or not, etc.), others lack some of these data. Concentration decrease during EVs storage at +4°C has been previously reported [25] with losses around 20% at 7 days and around 65% at 28 days. Much slower kinetics of losses compared to our results could be explained by larger vesicle size and lower diffusion coefficient. Concentration decrease of around 20% at 2–4 days and 40% for 15–25 days was reported [18] for vesicles with a mean diameter of 140–150 nm. However, these data should be treated with caution because EV samples were diluted by PBS for NTA measurements and filtered through 0.22-μm filters prior to size and concentration measurements. Several studies reported that EVs bind to the membrane during filtration to some extent [29,52,53], so filtration should be strongly avoided for highly diluted NTA samples as it could alter both the PSD and concentration.

The size increase was reported for EVs stored for 4 days at +4°C [17]. However, these data could not be directly compared to our results because the stored sample was additionally subjected to re-pelleting by centrifugation (100 000 g for 2 h) before size measurements. This additional re-pelleting might alter the mean particle size towards larger values for stored vesicles. Long term storage of vesicles (up to 25 days) has been shown to result in pronounced size decrease [14,18]. The nature of this effect could be proposed from TEM data reported in supporting information of the study [34]. EV samples, stored for 4 weeks at +4°C, contained vesicular debris, which allowed attributing the observed size decrease to the decomposition of vesicles. Thus, based on published data, the most likely cause of non-adsorptive losses in our study is vesicle decomposition. This hypothesis provides an explanation for the observed size divergence at longer storage times. Adsorption and decomposition influence the mean size in opposite ways, and small random deviations in their ratio for different samples might cause the variations in mean size.

### Efficiencies of different approaches for adsorption prevention

**Fig 5:**
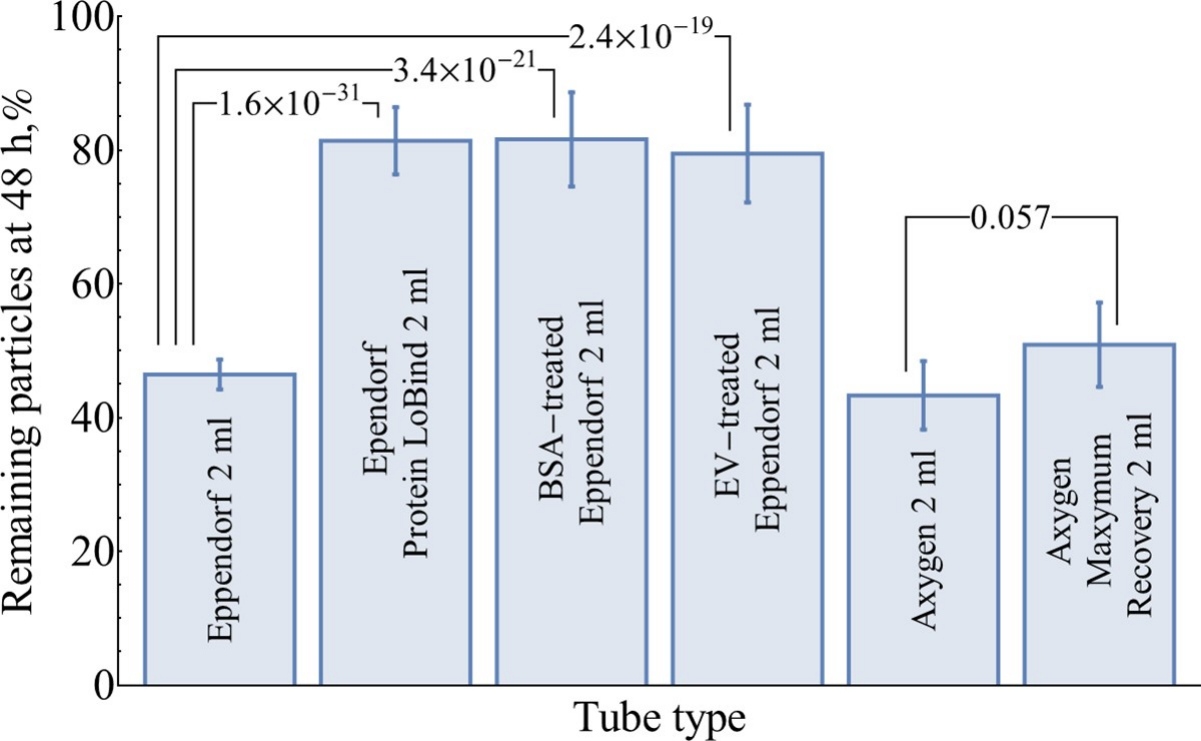
Comparison of total concentration losses for different types of tubes and tube treatments at 48 h of storage at +4°C. Error bars represent 95% CI of the mean (N = 108 for the ordinary Eppendorf tubes, N = 54 for the Eppendorf Protein LoBind tubes, N = 18 for the rest). *P*-values were calculated using a two-tailed unpaired t-test.

A widely practically used strategy to prevent non-specific binding is blocking the surface with an excess of protein or other reagent. Also, Eppendorf is not the only plasticware manufacturer who suggests a ‘low bind’ version of test tubes. Losses at 48 h of storage at +4°C were compared for 6 types of surfaces (Fig 5 and S4 Fig). Ordinary Eppendorf 2 ml tubes and Eppendorf Protein LoBind tubes were used as references. Tubes with surface blocked by BSA or EVs have shown losses of 18±7% and 21±7% correspondingly. Ordinary Axygen 2 ml tubes have shown losses similar to ordinary Eppendorf 2 ml (57±5% vs 53±2%). Surprisingly, Axygen Maxymum Recovery 2 ml tubes showed almost no improvement (50±6% losses at 48 h) over the ordinary Axygen tube.

These findings might be used as a guideline for reducing adsorption losses of purified EV during storage. Substantial losses in ordinary tubes (Eppendorf or Axygen) are strongly reduced for BSA-blocked tubes and Eppendorf Protein LoBind tubes. At the same time, not every tube stated as ‘low binding’/‘low retention’ is capable of reducing adsorption losses, as it has been shown for Axygen Maxymum Recovery tubes. Analogous products from other brands should be tested prior to use. The blockage of tube walls by EVs has been shown to be effective as well. This option could not be recommended for widespread use due to the high consumption of EVs for blockage procedure. Nevertheless, it could be the option for proteomic studies when the presence of additional proteins, e.g., BSA is undesirable, and also, some protocols recommend ‘to avoid all kinds of low-bind plastics’ [54].

### Purified EVs dissolved in 100 000 g supernatant are more stable than in PBS or DMEM

**Fig 6:**
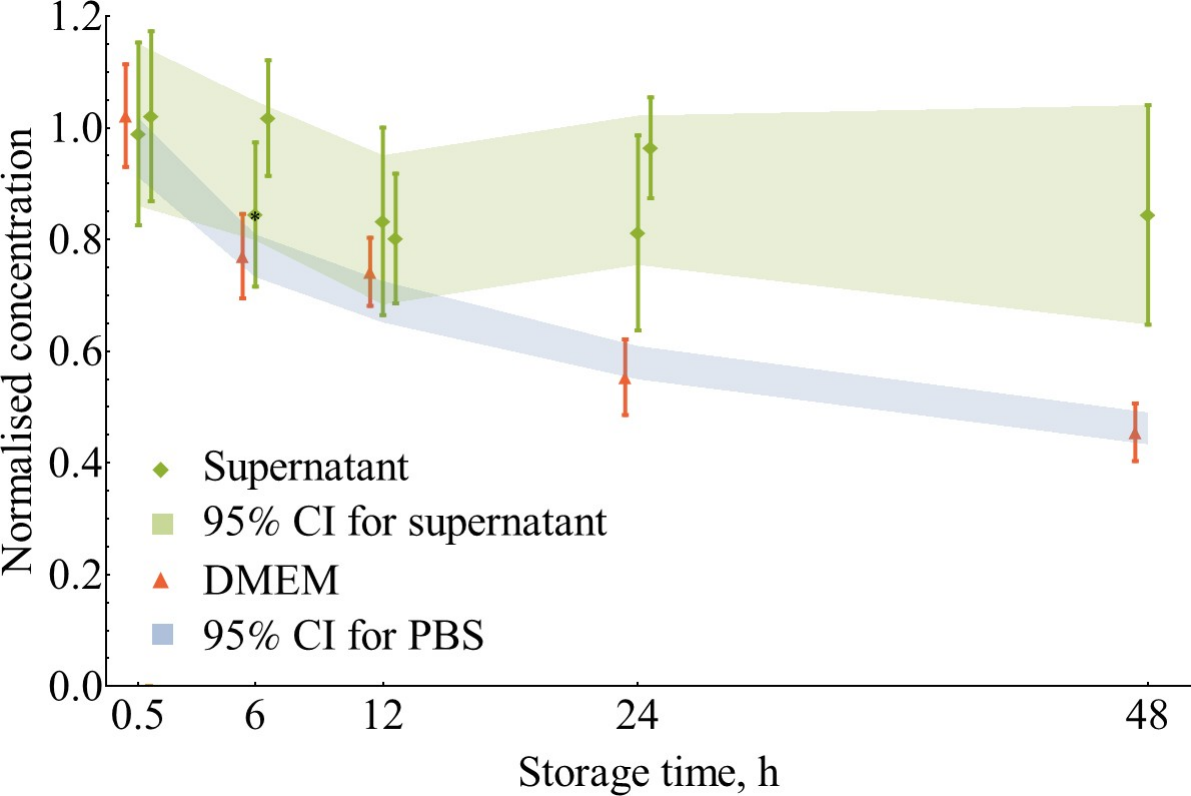
Storage of isolated EVs (0.5 ml, Eppendorf 2 ml tubes, +4°C) in the supernatant after 100 000 g centrifugation. EVs in the supernatant as a model of a conditioned medium were compared to EVs stored in serum-free DMEM. Ordinary Eppendorf 2 ml tubes were used. Error bars for individual data points represent 95% CI of the mean (N = 15 for point marked with an asterisk and N = 18 for the rest). Confidence interval (95%, N = 108) for storage in PBS was plotted for reference.

In order to provide additional insight into EVs behaviour in solutions at +4°C, we have studied one case of a complex medium. Direct comparison of losses for raw conditioned medium and purified vesicles in PBS is not straightforward due to the difference in initial vesicle concentrations and their state. EVs in raw conditioned medium might be considered as undisturbed, whilst vesicles purified with ultracentrifugation, then frozen, and thawed could be aggregated and deformed to some extent. To overcome this issue, in this experiment, we used a model system of purified vesicles dissolved in the supernatant after 100 000 g centrifugation. This medium contains residual particles at a concentration of (0.15±0.02) × 10^10^ particles/ml. This value was subtracted as blank. A concentration decrease of around 15% was observed during the first 12 h of storage and remained constant up to 48 h (Fig 6). On the other hand, EVs dissolved in DMEM, the main constituent of the serum-free medium used for cell cultivation, have shown the same kinetics as vesicles in PBS. This finding implies that the protective effect of supernatant is caused not by the DMEM components, but by some substances released by cells during conditioning. We explain this behaviour by *in situ* blockage of tube walls by free proteins present in conditioned medium in a manner similar to sometimes used resuspension of pelleted vesicles or dilution of the EV’s solution in PBS with the addition of BSA [55–59] or hydrophilic polymer [46].

## Conclusion

The present work provides experimental and theoretical evidences for the importance of adsorption prevention during +4°C storage/handling of purified EVs in PBS at concentrations below 2.1×10^10^ particles/ml. This range is typical for small and medium batches of vesicles, isolated for research purposes as well as EVs purified from small clinical samples. From the practical point of view, three solutions for loss reduction were found: Eppendorf Protein LoBind tubes and blockage of tube walls by either BSA or EVs. We also have shown that EVs might be rather stable at +4°C in ordinary tubes in conditioned medium, most likely due to *in situ* blockage of tube walls by free proteins released by cells during conditioning. The numerical simulation revealed two characteristic features of diffusion-controlled adsorption (if the sample is kept undisturbed): pronounced concentration decrease and size increase due to predominant losses of smaller vesicles.

## Supporting information

S1 Table The literature search for EVs stability at +4°C. (DOCX)

S2 Table Summary of S1 Table. (DOCX)

S1 Fig Comparison of normalised particle size distributions for fresh and frozen/thawed samples for two batches of EVs. (DOCX)

S2 Fig Alternative normalisation of data of Fig 3A by the concentration at 0.5 h for each curve. (DOCX)

S3 Fig Data of Fig 3D with no normalisation. (DOCX)

S4 Fig Comparison of total concentration losses for different types of 2 ml tubes and tube treatments during storage in PBS at +4°C, V = 0.5 ml. (DOCX)

S1 Appendix Numerical simulation of the diffusion-adsorption problem. (PDF)

S2 Appendix Study of inner walls for Eppendorf 2 ml ordinary tubes. (DOCX)
