# Supplementary material for: Adsorption of extracellular vesicles onto the tube walls during storage in solution
Source: PLoS One. 2020 Dec 28;15(12):e0243738. doi: 10.1371/journal.pone.0243738 (PMC7769454; doi:10.1371/journal.pone.0243738)
Supplement: S1 Table — (DOCX) [file pone.0243738.s001.docx]

**S1 Table. The literature search for EVs stability at +4°C**

| **#** | **Complex medium (C) / Pure EVs (P)** | **Storage medium and conditions** | **Evaluated parameter(s)** | **Result** | **Comments** | **Ref.** |
| --- | --- | --- | --- | --- | --- | --- |
| 1 | C | Human urine, 24 hours at +4°C | Qualitative comparison of Western blot bands for TSG101, AQP2, angiotensin-converting enzyme, and PODXL | Stable | Three samples were compared: (a) fresh urine, (b) stored at -80°C and thawed, (c) stored for 24 hours at 4°C, then frozen at -80°C and thawed. | Cheruvansky 2007 |
| 2 | C | Human blood serum, up to 96 hours at +4°C | Exosomal miRNAs (miR-21, miR-200b, miR-205) levels | Stable | Data from Fig 5A | Taylor 2008 |
| 3 | P | Exosomes, isolated from cell culture supernatants of HEK 293T cells, ECFCs and MSCs by ultracentrifugation, resuspended in PBS and stored up to 600 hours at +4°C | Mean particle size by Nanoparticle Tracking Analysis (NTA) | A decrease in size. Statistically significant after 72-96 hours |  | Sokolova 2011 |
| 4 | C | Rat cardiac myocyte exosomes, isolated by ExoQuick and resuspended in PBS,  18 hours at +4°C or +25°C | Qualitative comparison of Western blot bands for HSP60 and GAPDH for pelleted exosomes and supernatant after centrifugation at 164 000 g for 2 h (i.e. HSP60 and GAPDH release from exosomes). | Stable (no HSP60 or GAPDH release) | Only 4 and 25°C storage temperatures were analyzed. | Malik 2013 |
| 5 | P | Same exosomes, isolated by ultrafiltration followed by ultracentrifugation into sucrose cushion and resuspended in PBS,  18 hours at +4°C or +25°C |  | 50% of HSP60 is released from exosomes |  |  |
| 6 | C | Human blood plasma, 10 days at +4°C | TSG101-free plasma has been spiked with TSG101 containing exosomes.  Qualitative comparison of Western blot bands for TSG101 protein, found in luminal compartment of exosomes | Stable | Only 10 days of storage data were analysed because 30 and 90 days at +4°C were considered as long-term storage. | Kalra 2013 |
| 7 | C | Human blood serum, up to 24 hours at +25°C | Exosomal miRNA (miR-21, miR-142-3p) levels | 2-fold decrease | miR-1, miR-16, miR-122 have been excluded as they are not exclusively associated with EVs. | Koberle 2013 |
| 8 | C | Human blood plasma, 2 weeks at +4°C | Exosomal miRNA (miR-24, miR-181a, miR-451) C_t_ values after isolation of EVs from plasma with ExoQuick™ | Stable | MiR-16 has been excluded as it is not exclusively associated with EVs. | Ge 2014 |
| 9 | C |  | Exosomal miRNA (miR-24, miR-181a, miR-451) C_t_ values in plasma | Mean C_t_ increase of around 1.5-fold |  |  |
| 10 | P | Large EVs (up to 600 nm) isolated by centrifugation and resuspended in HBSS,  1, 7 and 28 days at +4°C or +20°C | EV counts by flow cytometry and antibacterial effect | Decrease both in number and biological effect. At 20°C, the decrease was significant with p<0.05 at 7 days storage, at +4°C – at 28 days storage. | Only +4°C and +20°C data were analysed. | Lorincz 2014 |
| 11 | C | Human blood plasma, up to 48 hours at +25°C | Exosomal miRNAs (let-7a, miR-142-3p) C_t_ values | Stable | Data were taken from Supplementary info Fig.2 | Enderle 2015 |
| 12 | P | Human blood plasma exosomes isolated by ultracentrifugation and resuspended in PBS. Overnight storage at +4°C | CD9 and CD63 levels measured by ELISA | Decrease of CD9 and CD63 by factors of 2.5 and 2.2 respectively | Protein low binding tubes were used for the storage of exosomes from conditioned medium. It is not clear whether they have also been used for plasma exosomes or not. | Zarovni 2015 |
| 13 | P | Blood plasma exosomes isolated by ultracentrifugation and resuspended in PBS, 4 and 8 weeks at +4°C | Particle size distribution (PSD) measured by DLS, semi-quantitative comparison of Western blot bands for CD63 and TSG101 proteins | Significant changes in PSD, a significant decrease in CD63 and TSG101 levels. | Data were taken from Supplementary info Fig.1 | Baranyai 2015 |
| 14 | C | Human clinical  cerebrospinal fluid (CSF), storage for 1 and 7 days at room temperature (RT) | EVs from stored samples were isolated by differential centrifugation. Particle counts, mean size, and RNA content were evaluated. | Stable. No significant changes at all measured parameters. | Only RT data were analysed. | Akers 2016 |
| 15 | C | Human serum from patients with metastatic colorectal cancer, stored for 24, 72, 168 h at +4°C and 6, 12, 24, 48 h at RT. | EVs were isolated from human serum using ExoQuick™ or PureExo® Exosome Isolation Kit. Qualitative comparison of Western blot bands for CD63 and TSG101. Measurement of total DNA concentration. | Stable. No significant changes in total DNA content. No visible signs of CD63 and TSG101 bands intensities changes. |  | Jin 2016 |
| 16 | C | Human saliva with cell debris and bacteria removed by centrifugation at 8000×g for 15 min at RT. Samples were stored for 7 and 28 days at +4°C | Total protein, dipeptidyl peptidase IV (DPP IV) activity, gel chromatography elution profiles, exosomes morphology by TEM, protein PAGE electropherograms, DPP IV, CD9, Alix and Tsg101 bands intensity comparison by Western blotting. | Stable, although minor signs of some protein degradation might be found on PAGE. | Only 7 days of storage data were analysed because 28 days at +4°C were considered as long-term storage. | Kumeda 2017 |
| 17 | P | Exosomes isolated from human saliva with gel chromatography, concentrated and stored in PBS for 20 months at +4°C | Gel chromatography elution profiles, exosomes morphology by TEM, protein PAGE electropherograms, DPP IV, CD9, Alix and Tsg101 bands intensity comparison by Western blotting. | Stable, although minor signs of some proteins cleavage might be found on PAGE. |  | Kumeda 2017 |
| 18 | P | Exosomes isolated from mouse bronchoalveolar lavage fluid (BALF) by differential ultracentrifugation and resuspended in PBS. Storage for 4 days at +4°C. | Mean hydrodynamic diameter measured by DLS, zeta-potential, protein content, including leaking/dissociating proteins, identified by label-free LC-MS/MS. | Change in zeta-potential from between −34.8 and −32.4 mV for a fresh sample to around -20 mV for stored one. | Changes in mean hydrodynamic size and proteomics of exosomes stored at +4°C were excluded from the comparison because the stored samples were subjected to additional pelleting/resuspension step. | Maroto 2017 |
| 19 | C | EVs in human urea stored for 7 days | Protein PAGE electropherograms, EVs count with NTA after isolation with differential ultracentrifugation. | Stable | NTA EV concentration data for this experiment was not presented in the paper. Though, the absence of differences in particle count was stated in the text. | Liu 2018 |
| 20 | P | EVs from the culture medium of human umbilical vein endothelial cells, infected by Kaposi’s sarcoma-associated herpesvirus.  EVs were stored at +4°C for up to 25 days. | Particle size and concentration measured by NTA, qualitative comparison of Western blot bands for CD63 and CD81. | A decrease in particle concentration |  | Park 2018 |
| 21 | C | Exosomes from HEK 293T conditioned media isolated by ExtraPEG method. Stored for 7, 15, and 30 days at +4°C in PBS | Qualitative comparison of Western blot bands for ALIX, HSP70, and TSG101. | A decrease in bands intensity. | Only data from Fig. 1C were used. Other data (Fig 1A, 1B, 2B) do not contain the fresh sample for comparison. | Cheng 2019 |
| Papers, excluded from comparison  These papers were excluded from the analysis because EVs stability conclusion cannot be drawn with the presented experimental data. | | | | | | |
| 22 | P | EVs isolated from human urine with differential ultracentrifugation and resuspended in 200:l of isolation solution (10 mM triethanolamine/250 mM sucrose (pH 7.6); 0.5 mM PMSF; 1 μm Leupeptin).  They were stored for 1h at +4°C, 1 week at -20°C or -80°C. | Total protein level, protein 1D SDS/ PAGE electropherograms, qualitative comparison for NHE3, TSG101, ALIX, and AQP2 band intensities by western blot |  | Excluded from the table as no fresh control was used. Thus there is no sample to compare to the one stored for 1 h at +4°C. | Zhou 2006 |
| 23 | C | Human urine stored for 2 h/1 day at +4°C or RT | Total particle concentration for 20-100 nm range using NTA. | More than a 2-fold decrease in concentration during 2 h or 1-day storage at either +4°C or RT. | Unfortunately, it is not clear whether scattering mode or QD labeling mode was used. If latter, it’s not clear which antibodies were used: CD24 or aquaporin 2.  Although the Methods section claims that every storage condition was tried both with proteinase inhibitor and without, the data of Fig. 6 contain +4°C and RT conditions without inhibitor only. Thus, there is no way to understand whether the concentration decrease was caused by EVs instability or proteinases. | Oosthuyzen 2013 |
| 24 | -- | Urine exosomes pellet after 200,000 g ultracentrifugation, 24 hours at +4°C | Exosomal miRNA (miR-200c) Ct value | Stable | Two samples were compared: (a) stored at -80°C and thawed, (b) stored for 24 hours at +4°C, then frozen at -80°C and thawed.  This study was excluded from comparison as EVs were stored not in solution, but as a pellet. | Lv 2013 |
| 25 | -- | EVs isolated from HEK293 with ExoQuick™ and resuspended in Radioimmunoprecipitation assay (RIPA) buffer.  30 minutes at +4°C, RT, and +37°C.  10 days at the same temperatures | HSP70, CD63 and CD9 levels, total protein levels, total RNA | At 30 minutes, no significant differences were found between +4°C, RT, and +37°C.  At 10 days all measured parameters decreased significantly compared to -70°C storage conditions | Results were excluded from the table as long as RIPA buffer fully lyses EVs. Thus, this paper describes not EVs stability, but rather the stability of EV’s lysate. | Lee 2016 |
| 26 | C | Human plasma, stored for days at +4°C or RT | Semi-quantitative measurement of a total of 3 proteins (CD9, CD63, CD81) using protein microarrays. | Stable.  No significant differences were found between samples stored at +4°C or RT on the one hand and -20°C or -40°C on the other hand. | Platelets were not removed from plasma prior to storage. Thus, EV levels might have been affected in both ways (sorption or production) by platelets. | Baek 2016 |
| 27 | C | Buffalo milk, 24 hours at +4°C | Exosomal miRNA (miR-21) level | 2-fold decrease | It is not clear whether the milk whey or whole milk prior to cells and fat separation was used for 24h storage. Most likely, it was whole milk, as it’s implied by the Discussion section. In this case, these data cannot be used in our study, as fat droplets and cell could interact with EVs | Baddela 2016 |
| 28 | P | EVs isolated from saliva with differential ultracentrifugation and resuspended in PBS. Stored for 1 h at +4°C, 1 week at -20°C and 1 month at -80°C | Total protein level, qualitative comparison for CD63 band intensity by western blot |  | Excluded from the table as no fresh control was used. Thus there is no sample to compare to the one stored for 1 h at +4°C. | Kechik 2018 |
| 29 | P | EVs isolated from human umbilical vein endothelial cells conditioned medium by UC, followed by encapsulation of glucuronidase and additional purification by SEC (final medium is PBS)  Storage for 7 days at +4°C | Mean size by NTA and activity of encapsulated glucuronidase. | Some changes in measured parameters occurred, but they were not statistically significant due to the low sample size (n=3) | Some changes in measured parameters occurred, but they were not statistically significant due to the low sample size (n=3) | Richter 2019 |
