# Supplementary material for: Adsorption of extracellular vesicles onto the tube walls during storage in solution
Source: PLoS One. 2020 Dec 28;15(12):e0243738. doi: 10.1371/journal.pone.0243738 (PMC7769454; doi:10.1371/journal.pone.0243738)
Supplement: S2 Table — (DOCX) [file pone.0243738.s002.docx]

**S2 Table. Summary of S1 Table**

|  |  | **Stability** | | **Total** |
| --- | --- | --- | --- | --- |
|  |  | **Stable** | **Not stable** |  |
| **Medium** | **Complex** | **10** | **3** | **13** |
|  | **Purified EVs** | **1** | **7** | **8** |
| **Total** | | **11** | **10** | **21** |
