# Supplementary material for: Adsorption of extracellular vesicles onto the tube walls during storage in solution
Source: PLoS One. 2020 Dec 28;15(12):e0243738. doi: 10.1371/journal.pone.0243738 (PMC7769454; doi:10.1371/journal.pone.0243738)
Supplement: S1 Fig — (DOCX) [file pone.0243738.s003.docx]

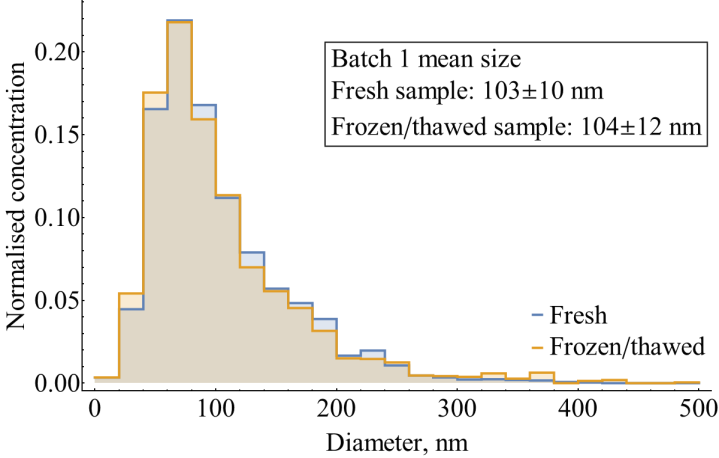

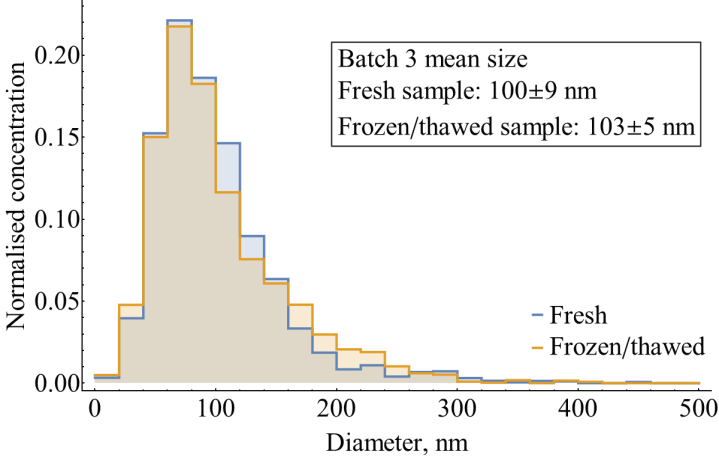


**S1 Fig. Comparison of normalised particle size distributions for fresh and frozen/thawed samples for two batches of EVs.**

Mean size is reported as 95% CI of the mean (N = 12).
