# Supplementary material for: Adsorption of extracellular vesicles onto the tube walls during storage in solution
Source: PLoS One. 2020 Dec 28;15(12):e0243738. doi: 10.1371/journal.pone.0243738 (PMC7769454; doi:10.1371/journal.pone.0243738)
Supplement: S2 Fig — (DOCX) [file pone.0243738.s004.docx]

**S2 Fig. Alternative normalisation of data of Fig 3A by the concentration at 0.5 h for each curve**.

As long as raw values at 0.5 h are represented as CI 95%, confidence intervals for ratios were calculated by bootstrapping procedure using empirical distributions of concentration from individual measurements. Error bars for individual data points represent 95% CI of the mean (N = 12 for point marked with an asterisk and N = 18 for the rest).
