# Supplementary material for: Adsorption of extracellular vesicles onto the tube walls during storage in solution
Source: PLoS One. 2020 Dec 28;15(12):e0243738. doi: 10.1371/journal.pone.0243738 (PMC7769454; doi:10.1371/journal.pone.0243738)
Supplement: S3 Fig — (DOCX) [file pone.0243738.s005.docx]

**S3 Fig. Data of Fig 3D with no normalisation.**

Evolution of particle concentration for three independent EV batches during storage in PBS (initial concentration 1×10^10^ particles/ml) in ordinary Eppendorf 2 ml tubes and Eppendorf Protein LoBind 2 ml tubes. Error bars for individual data points represent 95% CI of the mean (N = 12 for points marked with an asterisk and N = 18 for the rest). Numbers below groups indicate *p*-values of a Mann–Whitney test between the common tube and LoBind one for each time.
