# Supplementary material for: Adsorption of extracellular vesicles onto the tube walls during storage in solution
Source: PLoS One. 2020 Dec 28;15(12):e0243738. doi: 10.1371/journal.pone.0243738 (PMC7769454; doi:10.1371/journal.pone.0243738)
Supplement: S4 Fig — (DOCX) [file pone.0243738.s006.docx]

**S4 Fig. Comparison of total concentration losses for different types of 2 ml tubes and tube treatments during storage in PBS at +4°C, V = 0.5 ml.**

Error bars for individual data points represent 95% CI of the mean (N = 18). 95% CIs for ordinary Eppendorf tubes and Eppendorf Protein LoBind are plotted as a reference.
