# Supplementary material for: Adsorption of extracellular vesicles onto the tube walls during storage in solution
Source: PLoS One. 2020 Dec 28;15(12):e0243738. doi: 10.1371/journal.pone.0243738 (PMC7769454; doi:10.1371/journal.pone.0243738)

S1 Appendix. Numerical simulation of the diffusion-adsorption problem

1. The geometry of the problem

The geometry of Eppendorf 2 ml tube might be found on Eppendorf web site:

https://www.eppendorf.com/product-media/doc/en/140034_Technical-Data/Eppendorf_Consumables_Technical-data_Safe-Lock-Tube-20-mL_Safe-Lock-20-mL-technical-drawing.pdf

The inner radius of the tube is 0.44 cm. The lower part is close to half of the prolate spheroid with semi-axes 0.44, 0.44, and 0.68 cm.

Height of the cylindrical part of the tube now might be calculated from the total liquid volume of V = 0.5 cm^3, tube radius r_0 = 0.44 cm and long spheroid semi-axis a = 0.68 cm as follows:

$$h = \frac{\left(V - \frac{2\,\pi\, r0^2\, a}{3}\right)}{\pi\, r0^2} \approx 0.369 \text{ cm} \qquad (1)$$

The geometric area of contact between the tube wall and solution might be calculated as a sum of the areas for side wall of the cylinder (height h, radius r_0) and half of the area of the prolate spheroid (semi-axes of r_0, r_0 and a):

$$S = 2\,\pi\, r_0\, h + \pi\, r_0^2 \left(1 + \frac{a}{r_0 \sqrt{1 - \frac{r_0^2}{a^2}}} \times \arcsin\left(\sqrt{1 - \frac{r_0^2}{a^2}}\right)\right) \approx 2.697 \text{ cm}^2 \qquad (2)$$

2. Details of numerical simulation

Fick's second law describes a non-stationary diffusion problem and might be written in vector form as follows:

$$\frac{\partial C(\vec{r},t)}{\partial t} = D\, \Delta \vec{r} \qquad (3)$$

Test tube naturally has rotational symmetry, so cylindrical coordinates are most appropriate for this problem:

$$\frac{\partial C(r,z,t)}{\partial t} = D \left(\frac{\partial^2 C(r,z,t)}{\partial r^2} + \frac{1}{r}\frac{\partial C(r,z,t)}{\partial r} + \frac{\partial^2 C(r,z,t)}{\partial z^2}\right) \qquad (4)$$

This form is independent of rotational angle φ. Thus, the system is two-dimensional, which strongly reduces the computation time. Also, this approach reduces the problem to half of the tube section.

For correct usage of the Finite Elements method in Mathematica 10.2, a proper mesh should be created. As long as most differences are expected near tube walls, the mesh should be more frequent in these regions. The mesh with 70×70 elements in the cylindrical part and 70×100 elements in semi-elliptical part was created:

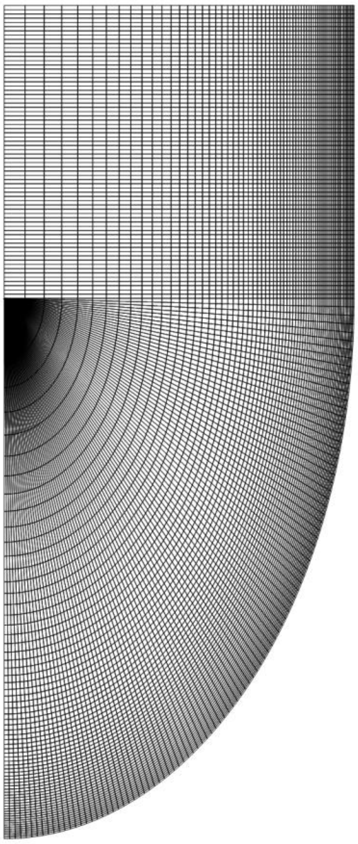

We might check the mesh coverage by $\iiint r dr d\varphi dz = 0.499983\ cm^3$, which is only 0.003% less than the desired volume of 0.5 cm^3.

Initial conditions might be stated as follows:

$$C(0, r, z) = \begin{cases} 0 \text{ for } z \geq 0 \text{ and } r \geq r0 \\ 0 \text{ for } z < 0 \text{ and } r^2/r_0^2 + z^2/a^2 \geq 1 \\ \text{else } C0 \end{cases} \quad (5)$$

For the case of unlimited adsorption concentration on the wall-solution boundary always equals to 0 due to immediate adsorption of any particles. Dirichlet boundary condition might be stated as:

$$C(t, r, z) = \begin{cases} 0 \text{ for } z \geq 0 \text{ and } r \geq r0 \\ 0 \text{ for } z < 0 \text{ and } r^2/r_0^2 + z^2/a^2 \geq 1 \end{cases} \quad (6)$$

NDSolve built-in function in Mathematica 10.2 automatically handles zero flux for all boundaries (left and top), where boundary conditions were not stated.

A full listing of the Mathematica 10.2 notebook used for simulation is provided at the end of this Appendix.

An example of concentration simulation in the described system for particle diameter = 100 nm with corresponding diffusion coefficient at +4°C D = 2.65×10^{-8} cm^2/s is shown in 3D and 2D contours for 1 h, 12 h, 24 h, and 48 h below.

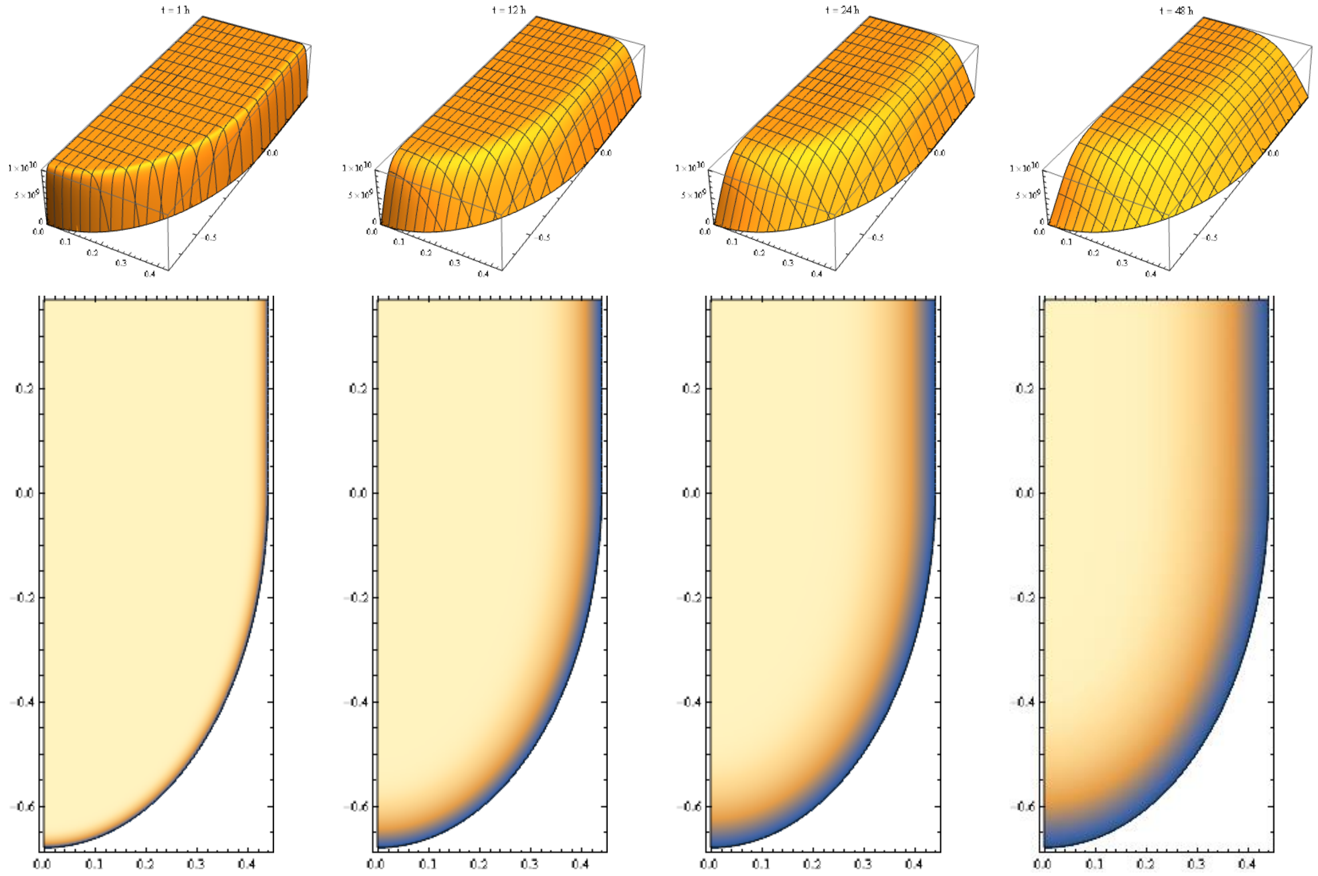

Simulated concentration profile for 0.5 ml of EVs with a diameter of 100 nm at +4°C at different storage times (from left to right): 1 h, 12 h, 24 h, 48 h.

In order to obtain the mean concentration over the entire tube at the desired times, the ratio of two numerical integrals was calculated for each time: $\iiint \mathrm{C}(t,r,z) \times r\, drd\varphi dz / \iiint r\, drd\varphi dz$

Size evolution was estimated as follows: the experimental histogram of PSD for the EV sample with a bin width of 20 nm was used. For every bin, the mean diameter was used (e.g., for the bin from 80 to 100 nm particles of the mean diameter of 90 nm were simulated) and a concentration for particles from this bin. Assuming the independence of diffusion for particles form different bins, the evolution of remaining concentrations was calculated for every bin. The resulting PSDs for every desired time were processed to calculate the mean diameter and the total concentration of particles of all sizes.

The simulated evolution of PSD and concentration for a sample of EVs in PBS in an ordinary Eppendorf tube is shown in the figure below.

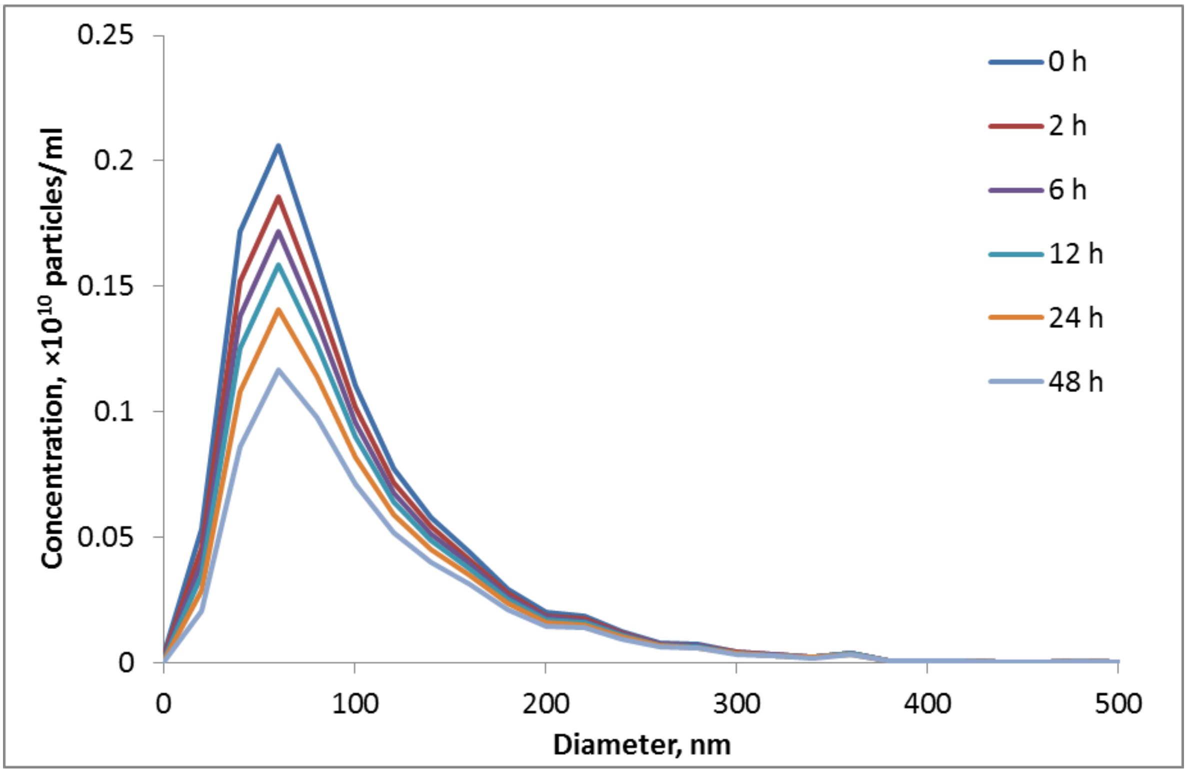

Simulated evolution of PSD and concentration for a sample of EVs (d_{mean} = 104 nm, 0.5 ml, +4°C) in PBS in an ordinary Eppendorf tube.

In[1]:=
```
T = 4; (* Temperature in deg C *)
d = 100; (* Diameter in nm *)
η = Exp[-3.7188 + 578.919 / (T + 273.15 - 137.546)]; (* Vogel equation for water viscosity http://ddbonline.ddbst.de/VogelCalculation/VogelCalculationCGI.exe?component=
 Water *)
Diff = (1.3806485 (T + 273.15)) / (3 π η d) 10^-7 ;(* Diffision coefficient in cm^2/s *)
r0 = 0.44; (* Tube radius in cm *)
a = 0.68 ; (* Height of the semi-elliptical part *)
C0 = 1 × 10^10; (* Initial concentration in particles/cm^3 *)
Vt = 0.5; (* Initial volume in cm^3 *)
h = (Vt - (2 π r0^2 a)/3) / (π r0^2) ;(* Height of the cylindrical part in cm *)

Days = 2;
```

In[11]:=
```
S = π r0 (r0 + a^2/Sqrt[a^2 - r0^2] ArcSin[Sqrt[a^2 - r0^2]/a]) + 2 π r0 h (* Geometric area of the contact between tube wall and solution in cm^2 *)
```

Out[11]= 2.69662

In[12]:=
```
(* Mesh creation setups *)
<< NDSolve`FEM`
NCyR = 70; (* The number of partitions in r direction for cylindrical part *)
NCyZ = 70; (* The number of partitions in z direction for cylindrical part *)
NSphR = 100; (* The number of radial partitions for semi-elliptical part *)
koef = 0.3; (* Coefficient for adjustment of mesh thickening near the tube wall. If koef=10, then no thickening occurs. The smaller the value,
the finer mesh near the wall *)
GenF = r0 ((koef + 1) #)/(koef + #) &;
```

In[18]:=
```
(* Mesh for cylindrical part *)
CyCoord = Flatten[Table[{GenF[x], z}, {x, 0, 1, 1/(NCyR - 1)}, {z, 0, h, h/(NCyZ - 1)}], 1]; (* Coordinates for vertices in cylindrical part *)
CyInc = Flatten[Table[{j*NCyZ + i, j*NCyZ + i + 1, (j - 1)*NCyZ + i + 1, (j - 1)*NCyZ + i}, {i, 1, NCyZ - 1}, {j, 1, NCyR - 1}], 1];
(* Indices for vertices in cylindrical part *)
(* Mesh for semi-elliptical part *)
SphCoord = Flatten[Table[{-GenF[x] Sin[θ], -GenF[x] Cos[θ] a/r0}, {x, 1/(NCyR - 1), 1, 1/(NCyR - 1)}, {θ, 0, -π/2, -π/2/(NSphR - 1)}], 1];
(* Coordinates for vertices in semi-elliptical part *)
SphCoord = Prepend[SphCoord, {0, 0}]; (* Prepend with (0,0) *)
SphInc = Flatten[Table[{j*NSphR + i + 1, j*NSphR + i + 2, (j - 1)*NSphR + i + 2, (j - 1)*NSphR + i + 1}, {i, 1, NSphR - 1}, {j, 1, NCyR - 2}], 1];
(* Indices for Quad vertices in semi-elliptical part *)
SphTrInc = Table[{i + 1, i + 2, 1}, {i, 1, NSphR - 1}]; (* Indices for Triangle vertices in semi-eliptical part *)
SphSh = Length[CyCoord]; (* Shift of the indices of semi-eliptical part *)
```

```
FullMesh = ToElementMesh["Coordinates" → Join[CyCoord, SphCoord],
   "MeshElements" → {QuadElement[CyInc], TriangleElement[SphTrInc + SphSh], QuadElement[SphInc + SphSh]}, "NodeReordering" → True]; (* Creating the mesh *)
CyCoord =.; CyInc =.;
SphCoord =.;
SphInc =.;
SphTrInc =.;
SphSh =.;
```

```
In[26]:= IntVol = NIntegrate[r, {r, z} ∈ FullMesh] ;
2 π IntVol (* Volume of the mesh in cm^3 *)
FullMesh["Wireframe"]

Out[27]= 0.499983
```

Out[28]=

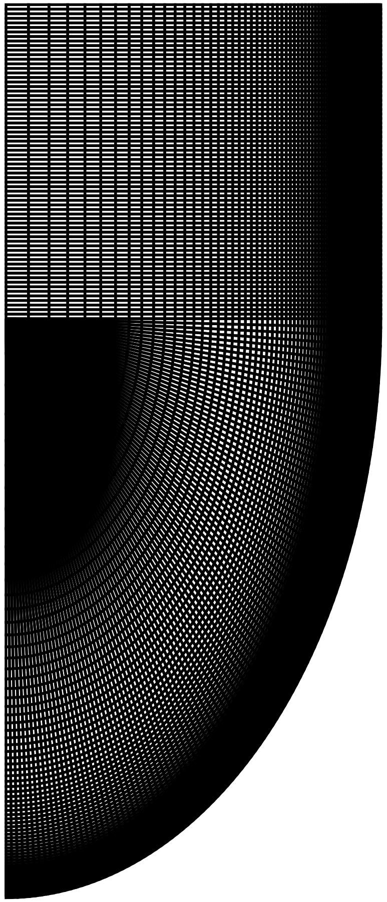

In[29]:=

```
(* Solve the differential equation *)
Off[NDSolve::femcsp]
sol = NDSolve[{
    ∂t Conc[t, r, z] == Diff (∂r,r Conc[t, r, z] + (∂r Conc[t, r, z])/r + ∂z,z Conc[t, r, z]),
    Conc[0, r, z] == Piecewise[{{0, z ≥ 0 && r == r0}, {0, z < 0 && r^2/r0^2 + z^2/a^2 == 1}}, C0],
    {DirichletCondition[Conc[t, r, z] == 0, z ≥ 0 && r == r0], DirichletCondition[Conc[t, r, z] == 0, z < 0 && r^2/r0^2 + z^2/a^2 == 1]}},
   Conc, {t, 0, Days*24*3600}, {r, z} ∈ FullMesh, MaxStepSize → 100, Method -> {"FiniteElement"}][[1]];
```

In[31]:=

```
(* Checking the total particle flux from t=0 up to t=Days for each point of the boundary *)
(* For this purpose we have to extract time mesh from the solution as well as coordinates for all boundary points *)
(* Then we have to define the normalized normal vector for each boundary point and calculate the concentration derivative along this vector *)
(* Then using the first Fick's law we might calculate the particle flux (particles/cm^2× s) for each boundary point and time *)
(* Finally, we shell integrate flux over the entire simulation duration for every boundary point and plot it as a function of z coordinate *)
Needs["DifferentialEquations`InterpolatingFunctionAnatomy`"];
PS = Conc /. sol; (* Extraction of pure interpolation function from the solution *)
MTimes = InterpolatingFunctionCoordinates[PS][[1]]; (* Extraction of time mesh used by NDSolve *)
RZCoord = FullMesh["Coordinates"]; (* Extraction of all (r,z) points from mesh *)
CyBordP = Cases[RZCoord, {r0, __}]; (* Selection of all boundary point for cylindrical part *)
ElBordP = Cases[RZCoord, {r_, z_} /; r^2/r0^2 + z^2/a^2 == 1 && r ≠ r0] ; (* Selection of all boundary point for semi-elliptical part  *)
CyNVec = Table[{1, 0}, Length[CyBordP]]; (* Calculation of normalized normal vectors for each boundary point of the cylindrical part *)
ElNVec = Table[Normalize[{a/r0 ElBordP[[i, 1]]/Sqrt[r0^2 - ElBordP[[i, 1]]^2], -1}], {i, Length[ElBordP]}];
(* Calculation of normalized normal vectors for each boundary point of the semi-elliptical part *)
(* https://www.khanacademy.org/math/multivariable-calculus/integrating-multivariable-functions/line-integrals-in-vector-fields-
  articles/a/constructing-a-unit-normal-vector-to-curve *)
CyFluxes = Table[0, Length[MTimes]];  (* Empty array for storage of fluxes for each time and each boundary point in the cylindrical part *)
ElFluxes = Table[0, Length[MTimes]];  (* Empty array for storage of fluxes for each time and each boundary point in the semi-elliptical part *)
Length[MTimes]
Dynamic[TSlice] (* Length of time mesh array together with dynamic counter to follow the progress of the next Do function *)
Do[ (* Loop over every time point in time mesh *)
  ConcP = PS[MTimes[[TSlice]], r, z]; (* Definition of Conc[r,z] at desired time *)
  RD = D[ConcP, r]; (* Definition of derivative D[Conc[r,z],r] *)
  ZD = D[ConcP, z]; (* Definition of derivative D[Conc[r,z],z] *)
  (* https://tutorial.math.lamar.edu/classes/calciii/directionalderiv.aspx *)
  CyFluxes[[TSlice]] = - Diff Table[With[{r = r0, z = CyBordP[[i, 2]]}, Evaluate[RD]], {i, Length[CyBordP]}];
  (* Calculation of flux for each boundary point in the cylindrical part *)
  ElFluxes[[TSlice]] = -Diff Table[With[{r = ElBordP[[i, 1]], z = ElBordP[[i, 2]]}, Evaluate[(RD ElNVec[[i, 1]] + ZD ElNVec[[i, 2]])]], {i, Length[ElBordP]}];
  (* Calculation of flux for each boundary point in the semi-elliptical part *)
  , {TSlice, 1, Length[MTimes]}];
ConcP =.; RD =.;
ZD =.;
```

Out[32]= 1777

Out[33]= 1777

In[36]:=
```
(* Now we might integrate over all times for each boundary point for both cylndrical and semi-elliptical parts *)
TotCyFl =
  Table[{CyBordP[[i, 1]], CyBordP[[i, 2]], NIntegrate[Interpolation[{MTimes, CyFluxes[[All, i]]}ᵀ, InterpolationOrder → 2][t], {t, MTimes[[1]], MTimes[[-1]]}]},
   {i, Length[CyBordP]}];
TotElFl =
  Table[{ElBordP[[i, 1]], ElBordP[[i, 2]], NIntegrate[Interpolation[{MTimes, ElFluxes[[All, i]]}ᵀ, InterpolationOrder → 2][t], {t, MTimes[[1]], MTimes[[-1]]}]},
   {i, Length[ElBordP]}];
TotFl = SortBy[Join[TotCyFl, TotElFl], #[[2]] &]; (* Joinig both parts together and sorting them by z coordinate *)
MTimes =.; CyBordP =.; ElBordP =.; CyNVec =.; ElNVec =.; CyFluxes =.; ElFluxes =.;
BindCap = N[1/(π (d 10^-7)^2)] (* Maximum binding capacity per cm^2 for the case of supported lipid bilayer *)
ListPlot[TotFl[[All, {2, 3}]], PlotRange → {All, All}, AxesLabel → {"z, cm", "Total particle flux over " <> ToString[Days * 24] <> " hours per cm^2"},
 ImageSize → Medium]
(* Plotting the result *)
```

Out[40]= 3.1831×10^9

Out[41]=
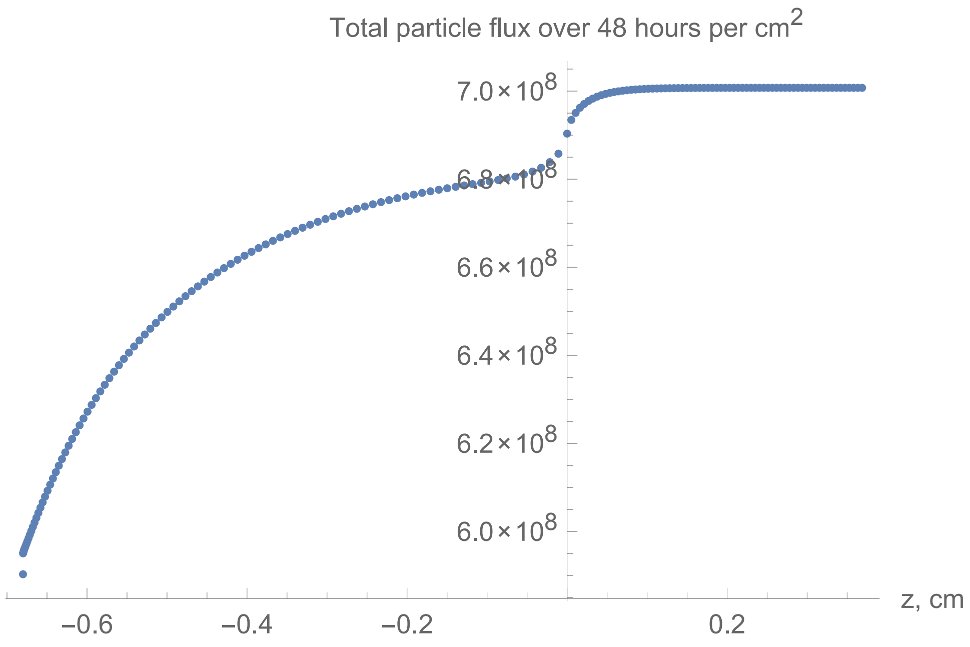

In[42]:=
```
(* Visualization of concentration profiles *)
TSection = 48; (* Visualization time in hours *)
VisTable = Table[{RZCoord[[i, 1]], RZCoord[[i, 2]], PS[3600 TSection, RZCoord[[i, 1]], RZCoord[[i, 2]]]}, {i, Length[RZCoord]}];
RZCoord =.; PS =.;
ListPlot3D[VisTable, PlotRange → {{0, r0}, {-a, h}, {0, 1.01 C0}}, PlotLabel → "t = " <> ToString[TSection] <> " h", BoxRatios -> {r0, a + h, r0 / 2}, ImageSize → Medium]
ListDensityPlot[VisTable, PlotRange → All, AspectRatio → (h + a) / r0, BoundaryStyle → Directive[Black, Thin], ImageSize → Medium]
VisTable =.;
```

Out[44]=

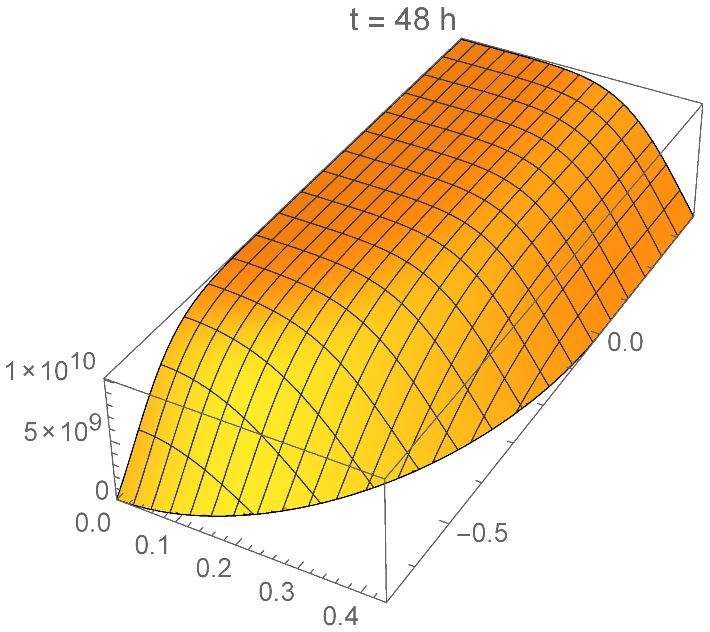

Out[45]=

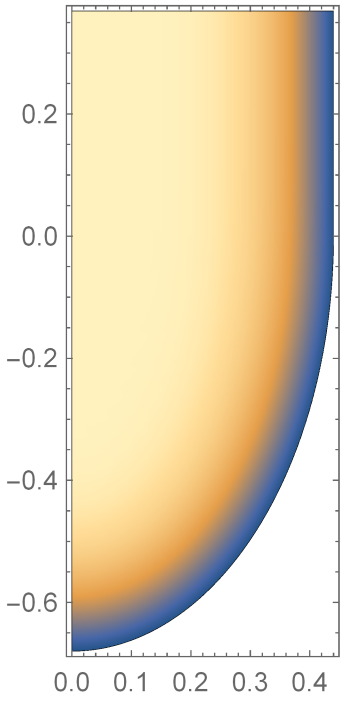

In[47]:=

```
(* Calculation of mean concentration over the tube at desired times *)
Dynamic[i] (* iterator to follow the progress *)
OutTimes = {0, 0.5, 1, 2, 4, 6, 12, 24, 48}; (* desired times in hours *)
ConcTable = Table[PS = With[{t = 3600 i}, Evaluate[Conc[t, r, z] /. sol]]; NIntegrate[PS r, {r, z} ∈ FullMesh] / IntVol, {i, OutTimes}];
(* Calculation of the mean concentration over the entire tube by integration *)
ConcTable / 10^10 // TableForm (* Outputs the result as table *)
CTPlot = Transpose[{OutTimes, ConcTable}]; (* Outputs the result as graph *)
Show[ListPlot[CTPlot, PlotRange → {{-1, Days * 24 + 1}, {0, 1.01 C0}}, Joined → True, DataRange → {0, Days * 24}, ImageSize → Large],
 ListPlot[CTPlot, PlotRange → {{0, Days * 24 + 0.2}, {0, C0}}, DataRange → {0, Days * 24}]]
```

Out[47]= 48

Out[50]//TableForm=

```
0.995899
0.958191
0.941287
0.917584
0.884534
0.859563
0.804466
0.729582
0.62983
```

Out[52]=

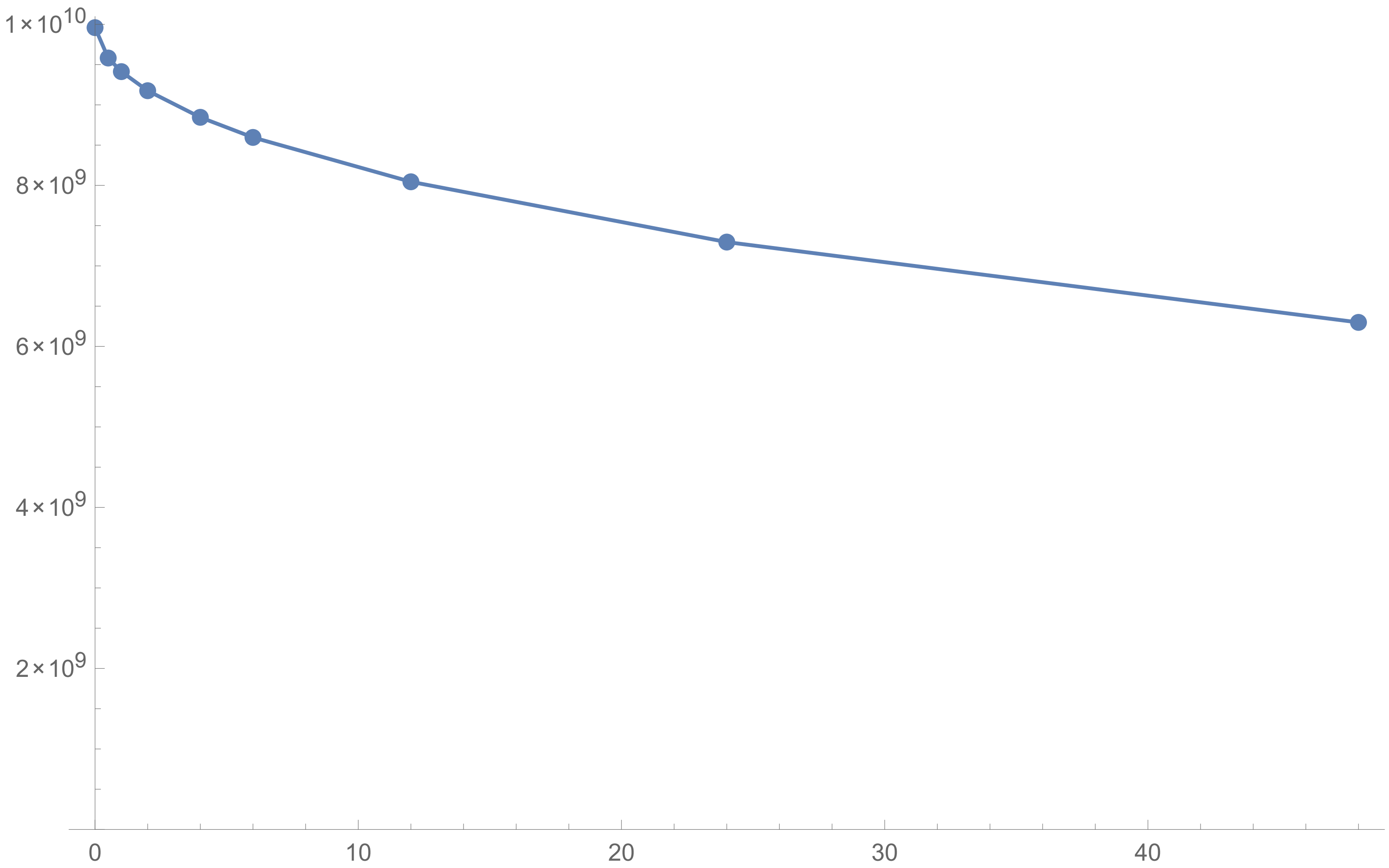

Supplement: S1 Appendix — (PDF) [file pone.0243738.s007.pdf]
