# Supplementary material for: Adsorption of extracellular vesicles onto the tube walls during storage in solution
Source: PLoS One. 2020 Dec 28;15(12):e0243738. doi: 10.1371/journal.pone.0243738 (PMC7769454; doi:10.1371/journal.pone.0243738)
Supplement: S2 Appendix — (DOCX) [file pone.0243738.s008.docx]

**S2 Appendix. Study of inner walls for Eppendorf 2 ml ordinary tubes**

*Methods*

Scanning electron microscopy (SEM). Four pieces of around 3×5 mm were cut from the lower part of the Eppendorf 2 ml ordinary tube. Survey images of the tube’s inner wall with ×500 magnification were obtained using a Hitachi TM3000 instrument (Hitachi, USA) in Charge-up Reduction Mode at 15 kV. In order to enhance the weak contrast characteristic of this mode 12 images of the same area were recorded and averaged using the Blend built-in function of Mathematica 10.2 package (Wolfram Research, USA).

Atomic Force Microscopy (AFM). Two pieces of around 2×5 mm were cut from opposite sides of the Eppendorf 2 ml ordinary tube. The inner surface of pieces was examined in PBS using Asylum MFP-3D-SA AFM (Asylum Research, USA) in tapping mode using fpN 10S (Super) cantilevers (State Research Institute for Problems in Physics named after F.V. Lukin, Russia) with tip curvature radius ≤ 10 nm, tip cone angle ≤ 22° and force constant of 5.5-22.5 N/m. Typical resonant frequency for used cantilevers on air was 190-200 kHz and 75-83 kHz in liquid.

*Results and discussion*

Representative survey SEM image of Eppendorf 2 ml inner tube wall (top) and image from the top camera of AFM microscope (bottom):


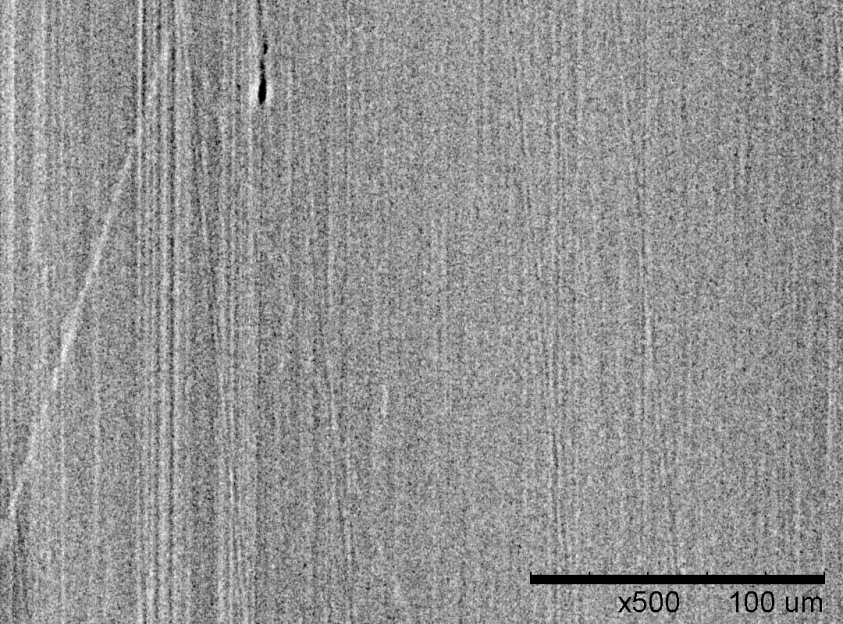

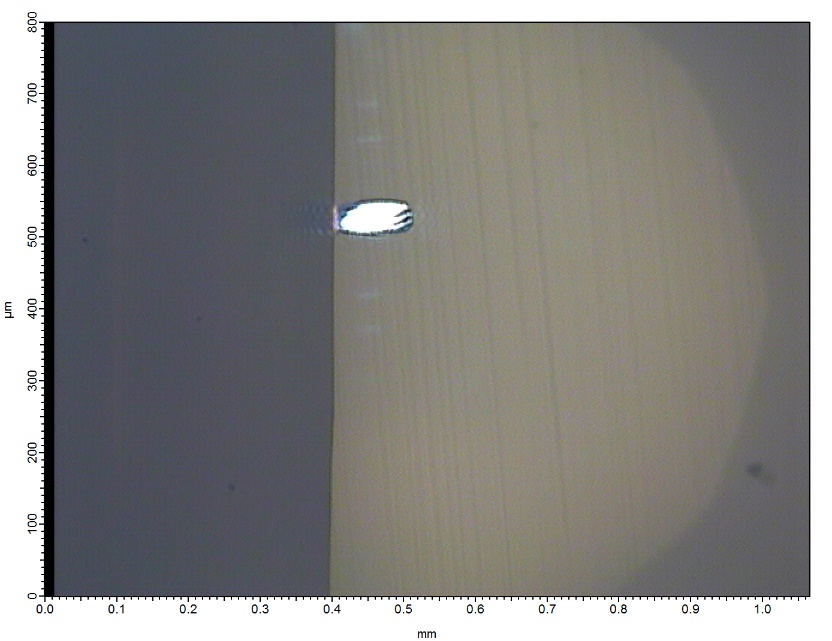


Representative AFM images of Eppendorf 2 ml inner tube wall in liquid


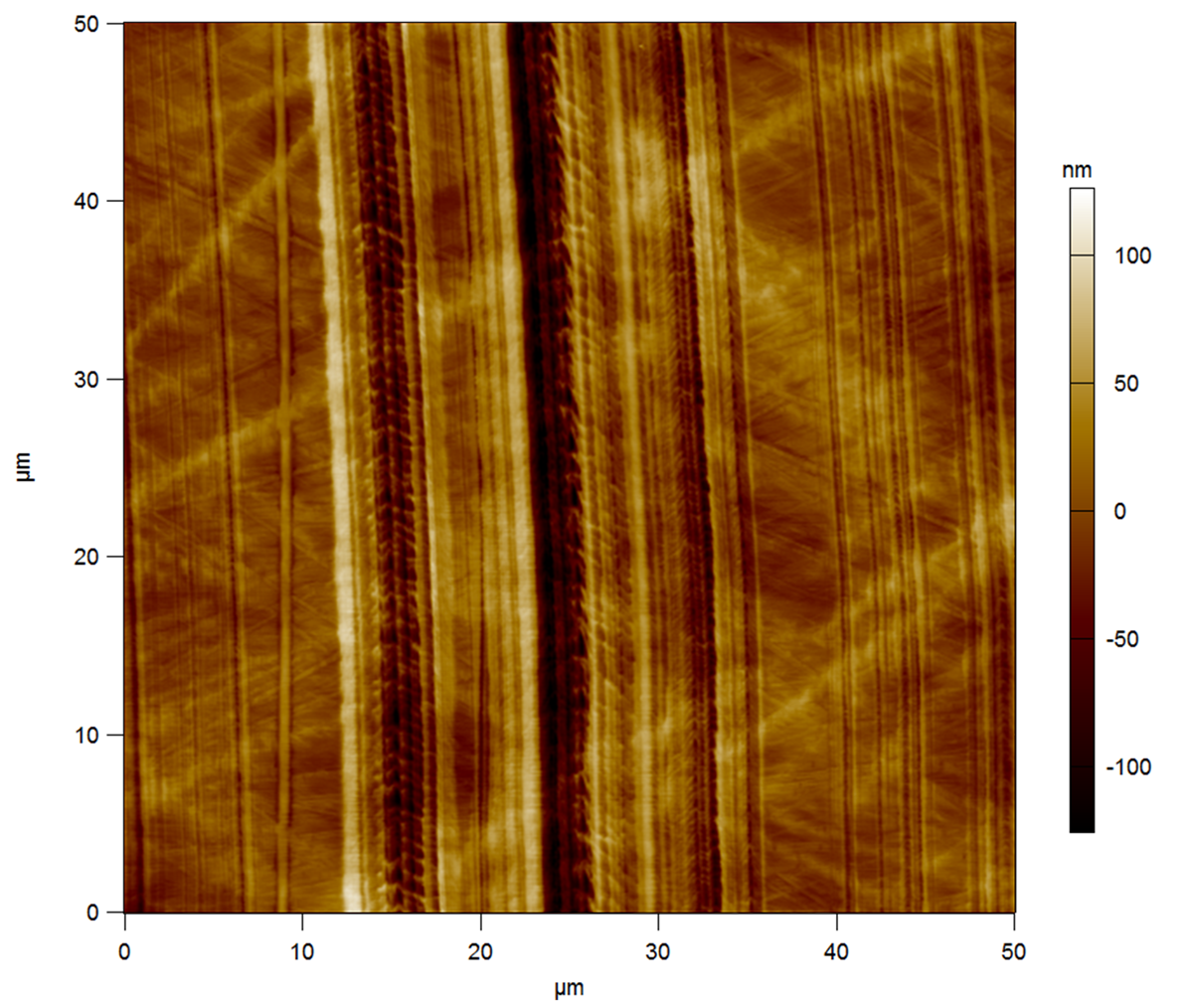

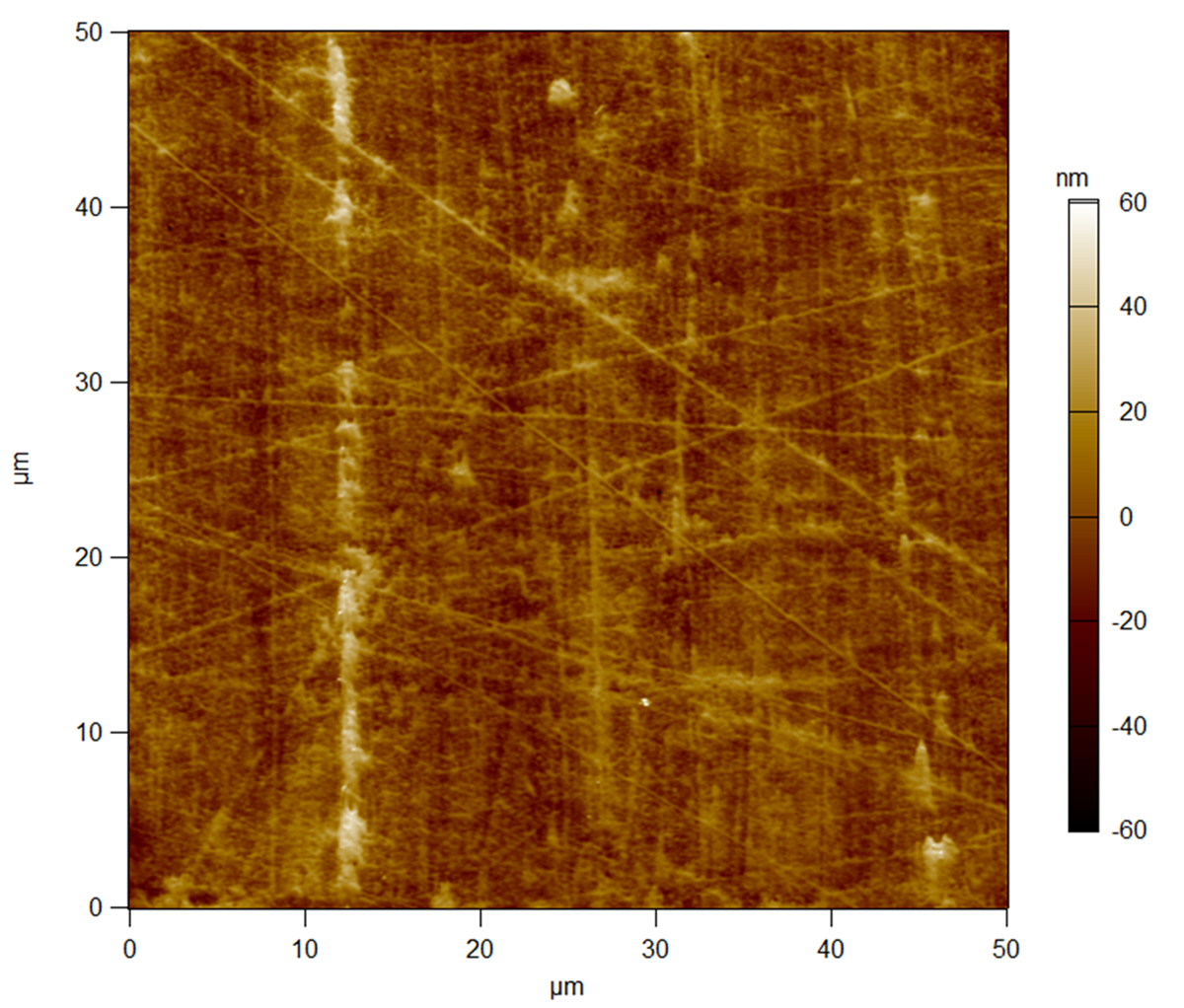


Survey SEM images showed the presence of linear objects on the surface, with most of them aligned vertically. In order to analyse the topography of the surface, 50×50 μm AFM images were obtained for 3 different tubes. Three types of objects originating from the production process were found:

a) Ridges of various orientations (look white on AFM images). They originate from scratches on the metal mould created by the polishing process. During injection moulding, these scratches created embossed lines on the surface of the tube.

b) Vertical scratches (top AFM image) of up to 200 nm deep. Pointing upwards features on the edges of the scratch show that it has been created by pulling a mould with protrusions out of the tube. These scratches were observed on 2 out of 3 studied tubes.

c) In one tube, no scratches were found. Instead of them, topography contained upwards scraped features (bottom AFM image). Most likely, they occur by pulling a mould out of the tube in the case of a smooth mould surface.

AFM images could be used to calculate the ratio of the real surface area to geometric one. Despite the deep scratches, the excess surface area was small, ranging from 0.07 - 0.15% for scraped tube up to 0.51-1.67% for scratched tubes.

Thus, found surface features might potentially affect the binding affinity of EVs (as long as binding inside the scratch is preferable), but not the area available for adsorption.
